# Visible light-controlled living cationic polymerization of methoxystyrene

**DOI:** 10.1038/s41467-022-31359-4

**Published:** 2022-06-24

**Authors:** Lei Wang, Yupo Xu, Quan Zuo, Haojie Dai, Lei Huang, Meng Zhang, Yongli Zheng, Chunyang Yu, Shaodong Zhang, Yongfeng Zhou

**Affiliations:** School of Chemistry and Chemical Engineering, Frontiers Science Center for Transformative Molecules and State Key Laboratory of Metal Matrix Composites, 800 Dongchuan Road, Shanghai Jiao Tong University, Shanghai, 200240 China

**Keywords:** Polymer synthesis, Polymerization mechanisms, Synthetic chemistry methodology

## Abstract

Photo-controlled living polymerization has received great attention in recent years. However, despite the great success therein, the report on photo-controlled living cationic polymerization has been greatly limited. We demonstrate here a novel decolorable, metal-free and visible light-controlled living cationic polymerization system by using tris(2,4-dimethoxyphenyl)methylium tetrafluoroborate as the photocatalyst and phosphate as the chain transfer agent (CTA) for polymerization of 4-methoxystyrene. This polymerization reaction under green LED light irradiation shows clear living characteristics including predictable molar mass, low molar-mass dispersity (*Đ* = 1.25), and sequential polymerization capability. In addition, the photocatalytic system exits excellent “on-off” photo switchability and shows the longest “off period” of 36 h up to now for photo-controlled cationic polymerization. Furthermore, the residual photo-catalyst is easily deactivated and decolored with addition of a base after the polymerization. The present study has extended the photo-controlled living cationic polymerization systems with new organic photocatalysts, phosphate CTA and polymerizable monomer as well as the new properties of excellent photostability and in-situ decolored capacity.

## Abstract (web summary)

Photo-controlled living polymerization has received great attention in recent years but the reports on photo-controlled living cationic polymerization are limited. Here, the authors demonstrate a metal-free and visible light-controlled living cationic polymerization using a photocatalyst and a phosphate as the chain transfer agent for the polymerization of 4-methoxystyrene.

## Introduction

In the last decade, considerable attention has been paid to living polymerizations mediated by external stimuli (mechanical, electro-chemical, etc)^1–5^. Among these stimuli, the use of light source is very attractive because of its great capacity on spatio-temporal control of the polymerization process, apart from the control of polymer chain length, low dispersity endowed by their living feature. For example, pioneered by Hawker and co-workers^6,7^, photo-induced atom transfer radical polymerization (photo-ATRP) has been quickly developed in the last few years, which employs a variety of photocatalysts including Ir(ppy)_3_^6^, perylene^8^, 10-methylphenothiazine^9^, *N*-aryl phenothiazine^10^, *N*,*N*-diaryl dihydrophenazine^11^, *N*-aryl phenoxazine^12^, dimethyl dihydroacridines^13^, oxygen-doped anthanthrene^14^, and other organic photocatalysts (OPCs) with complex structures^15–17^. Boyer et al. designed visible photo-induced electron transfer reversible addition fragmentation chain transfer (PET-RAFT) polymerization with various photocatalysts and thiocarbonylthio compounds as chain transfer agent (CTA), which showed excellent on-off switching of the living polymerization^18–25^. Ring-opening metathesis polymerization (ROMP)^26,27^ and ring-opening polymerization (ROP)^28–30^ regulated by visible light have also been reported.

**Fig. 1:**
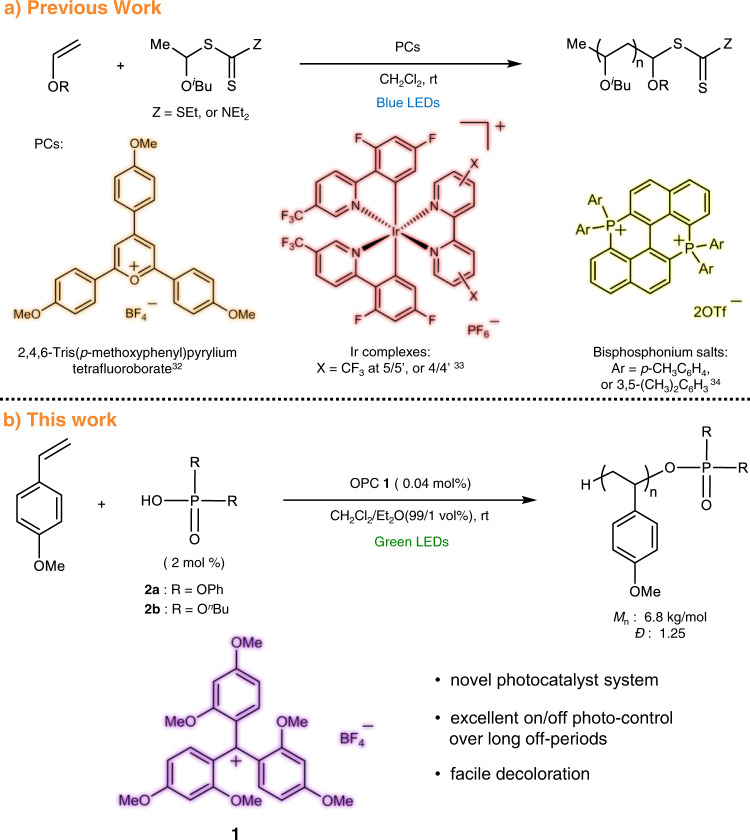
Recent reported in visible light driven photo-controlled living cationic polymerization. **a** Photocatalysts (PCs) and CTA employed in previous work. **b** The novel visible light-driven photo-controlled living cationic polymerization system in this work.

Up to date, the photo-regulated polymerization strategy of vinyl monomers has mainly focused on the living radical polymerization (LRP), while its application to living cationic polymerizations had surprisingly been overlooked with only few exceptions^31–34^. Especially, the photo-controlled living cationic polymerization, in which the living growth of polymer chain can be efficiently controlled under the “on-off” irradiation of light, has seldom been reported. Among them, Fors and coworkers pioneered it by using 2,4,6-tris(*p*-methoxyphenyl)pyrylium tetrafluoroborate (Fig. 1a) as the photoredox catalyst to synthesize poly(vinyl ether)s with controlled molar mass and low dispersity under irradiation of blue light-emitting diodes (LED)^32^. Aiming at improving the temporal control of this method, the same group employed more stable Iridium complexes to acquire excellent “on-off” switching of the chain growth (Fig. 1a)^33^. Liao and coworkers later realized photo-controlled living cationic polymerization of vinyl ethers by employing bisphosphonium salts as photocatalysts (Fig. 1a)^34^. These rare examples show the field of photo-controlled living cationic polymerizations is at its infancy, and the broadening of the scope of new photoredox catalysts and monomers is highly demanding^35,36^.

Furthermore, the reported photo-induced living polymerization systems generally suffer from the problems brought by residual photocatalysts. These residual photocatalysts trapped in the polymer matrix can cause color contamination^14^ or heavy metal toxicity if the catalysts are metal ion-loaded, which greatly limit the potential applications of these polymers in fields including advanced photoelectronic materials and biomaterials^37^. In addition, they will induce polymer degradation and side reactions due to strong oxidization of excited state photocatalysts under light. Although several methods such as column chromatography, precipitation, and centrifugation have been tried to eliminate the catalytic residual^38^, these procedures are often nontrivial and costly. Therefore, endeavor is also urgently required to readily remove or decrease residual photocatalysts.

In this article, we therefore report on a new photocatalytic system composed of OPC tris(2,4-dimethoxyphenyl)methylium tetrafluoroborate (**1**) and phosphate CTAs, so to realize visible light-controlled living cationic polymerization by using 4-methoxystyrene (*p*-MOS) as a proof-of-concept monomer. This reaction shows clear living characteristics including predictable molar mass, low molar-mass dispersity, and sequential polymerization capability. In addition, this polymerization system shows excellent photo “on-off” switching ability: the polymerization only occurs in a living way under light irradiation, while totally halts in the dark period up to 36 h, which is, to our knowledge, the record of dormant period up to now for photo-controlled cationic polymerization. Moreover, the erstwhile dark purple photocatalyst can be decolored and deactivated by simply reacting with an alkaline after the polymerization. Such a photocatalyst deactivation method circumvents the tedious decoloration and catalyst removal process in conventional photo-induced polymerizations.

## Results

### The photophysical and electronic properties of OPC 1

It has been previously demonstrated that triarylmethyl cations can undergo reversible redox processes via single electron transfer (SET)^39^. Among them, triphenylmethyl cation was extensively studied as very efficient activators and as one-electron oxidants for olefin polymerization reactions^40^. However, it hitherto has not been applied to visible photoinduced reaction, let alone living polymerizations. In the current study we demonstrate, for the first time, that it can serve as a powerful photocatalyst to achieve visible light-induced living cationic polymerization of *p*-MOS.

**Fig. 2:**
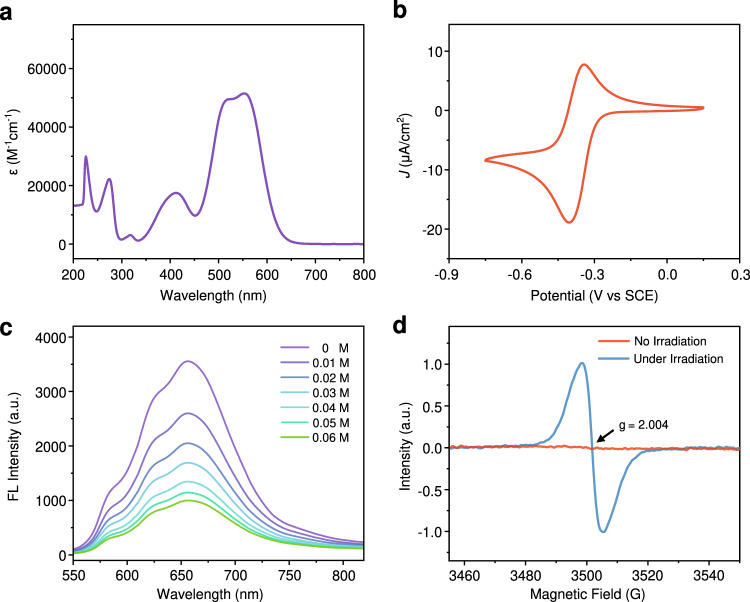
Characterizations of OPC 1 and photoelectron transfer between OPC 1 and CTA 2a. **a** UV absorption spectrum of **1**. **b** The cyclic voltammogram of **1**. **c** Fluorescence changes of photocatalyst **1** with the addition of **2a**. **d** Steady-state electron spin resonance spectrum of **1** with **2a** under 532 nm irradiation.

OPC **1** was prepared by one-pot synthesis as a dark purple solid in 83% yield (see synthetic details in Supplementary Information), which exhibits good solubility in common solvents like dichloromethane (CH_2_Cl_2_). Its UV/vis absorption spectrum shows a characteristic twin absorption band at *λ*_max_ = 517 (ε_max_ = 49 500 M^−1^cm^−1^) and 553 nm (ε_max_ = 51 000 M^−1^cm^−1^) in CH_2_Cl_2_, therefore allowing for the absorption of low-energy green light source (Fig. 2a). OPC **1** exhibits an excellent photochemical stability, as no change in absorption was observed after 12 h of 5 W green LED light irradiation (Supplementary Fig. 8). Its cyclic voltammogram shows a highly reversible redox wave under argon atmosphere, which corresponds to the transition from **1** to **1•** radical (Fig. 2b). It has a relatively lower ground state potential (E_1/2_ = –0.36 V vs. SCE) as compared to other reported methoxy-substituted triarylmethyl cations (Supplementary Table 1), and its excited state potential is at +1.55 V vs. SCE according to Eq. 1^41^.

1 $${E}_{{Ox}}[{{PC}}^{* +}/{{PC}}^{{{\bullet }}}]={E}_{{Red}}[{{PC}}^{+}/{{PC}}^{{{\bullet }}}]+{\varDelta E}_{{excit}}[{{PC}}^{+}/{{PC}}^{* +}]$$

The excitation energy, ${\varDelta E}_{{excit}}[{{PC}}^{+}/{{PC}}^{* +}]$ can be obtained from the fluorescence spectra (Fig. 2c). The lower potential is not only beneficial to the stability of the catalyst and monomer mixture in a dark environment, but also to the recapturing of propagating cationic species and deactivation of polymerization (*vide infra*). These photophysical properties of **1** therefore prompted us to attempt its application to photo-controlled living polymerization.

### The photoinduced electron transfer between OPC 1 and phosphate CTAs

Beside photocatalyst **1**, a suitable CTA is also necessary for photoinduced cationic living polymerization. The efficient CTAs for such purpose are so far limited to sulfur compounds^33,35,42,43^. Although these CTAs are widely used for chain transfer related reversible deactivation radical polymerization (RDRP), their unpleasant odor and residual color hurdle their large-scale applications^44^. In contrast, phosphate derivatives are a type of biochemical intermediates that are readily accessible, non-toxic, colorless and odorless. The Kamigaito group used phosphates as CTAs for cationic RAFT polymerization of vinyl ethers^45^. However, to our knowledge, they haven’t been used for photo-controlled living polymerization.

In the current study, diphenyl phosphate (**2a**) or dibutyl phosphate (**2b**) with predetermined quantities were first mixed with photocatalyst **1**, so as to ascertain its stability in the presence of nucleophilic P(O)O–R groups before irradiation^46^. The characteristic absorption of **1** was unambiguously observed without any change of absorption in visible region, suggesting no reaction occurred between **1** and phosphate CTA at the ground state (Supplementary Fig. 9–10).

The photoinduced electron transfer (PET) process between **1** and phosphate CTA was then monitored by steady-state fluorescence spectroscopy^47^. The fluorescence of **1** in CH_2_Cl_2_ was gradually quenched by addition of **2a**, **2b**, or *p*-MOS without formation of new emission band (Fig. 2c, Supplementary Fig. 11–12), indicating that PET can occur between the excited OPC **1** and either the CTA or the monomer. The rate constants of the fluorescence quenching of *k*_q_, was determined by Stern−Volmer equation (Eq. 2).

2 $${F}_{0}/F=1+{k}_{q}{\tau }_{{{{{{\bf{1}}}}}}}[Q]$$

where *F*_0_ is the fluorescence intensity before addition of quencher, *τ* is the fluorescence lifetime of the catalyst, and [*Q*] is the concentration of quencher. The *k*_q_ was determined to be 1.49 × 10^10^ M^−1^S^−1^ for **2a**, 1.17 × 10^8 ^M^−1^S^−1^ for **2b**, and 4.92 × 10^9 ^M^−1^S^−1^ for *p*-MOS, respectively. These results therefore revealed considerably efficient PET process between photocatalyst **1** and either the CTA or the monomer, among which phosphate **2a** is more potent. Besides, the bimolecular quenching constant *k*_q_ of **2a** at *ca*. 10^10 ^M^−1^S^−1^ suggest that this PET proceeds via a diffusion-controlled process^43^.

The fluorescence decay of **1** was also measured using 532 nm pulsed excitation, and its lifetime continuously decreased with increasing amount of **2a**, which also confirms the aforementioned PET process (Supplementary Fig. 14). Meanwhile, the electron spin resonance (ESR) spectroscopy was measured to further verify PET process between **1** and **2a** (Fig. 2d). When **1** was directly irradiated by green LED, no free radical signal was observed. In contrast, when mixed with **2a**, photocatalyst **1** under irradiation with 532 nm light generated an ESR signal with g = 2.004, corresponding to a free radical species that can be assigned to **1•** (Fig. 2d).

With the above-mentioned clues, we postulated that phosphate CTA **2a** or **2b** can be oxidized into a radical cation by photo-excited **1** via PET process, and their combination might provide a novel catalytic system for photo-controlled living cationic polymerization.

### Living polymerization of *p*-MOS triggered by visible light

As a proof of concept, the application of the novel photocatalytic system of CTA **2a** or **2b** and OPC **1** to visible light-induced living polymerization was tested with *p*-MOS as proof-of-concept monomer. The polymerization reaction was conducted at room temperature in a solvent mixture of CH_2_Cl_2_ and Et_2_O (99/1 vol%) under argon atmosphere, which was triggered with a light source of 5 W, 532 nm LED (Fig. 1).

As can be seen from Table 1 (entry 1), no reaction occurred in the absence of OPC **1**. Without **2a** or **2b** but only photocatalyst **1** (entry 2), the polymerization yielded the polymers with *Đ* = 1.65. It indicates OPC **1** can directly activate the monomer, leading to an uncontrolled polymerization^43^, which agrees with their oxidative potentials that will be discussed in detail in the mechanistic section (*vide infra*). On the other hand, when CTA **2a** or **2b** and photocatalyst **1** were both present the polymerizations progressed steadily (entries 3–10), as will also be discussed afterwards.

**Table 1:** Visible light-controlled living cationic polymerization of *p*-MOS^a^.

Entry	CTA	1:CTA:monomer	Time (min)	Conv. %	*M*_n_(exp)^b^(kg/mol)	*M*_n_(theo)^c^(kg/mol)	*Đ*^b^
1	**2a**	0:2:100	60	0	–	–	–
2	**-**	0.04:0:100	60	99	103.7	–	1.65
3	**2a**	0.08:2:100	40	98	6.9	7.2	1.29
4	**2a**	0.04:2:50	60	91	3.7	3.4	1.27
5	**2a**	0.04:2:100	60	87	7.1	6.4	1.25
6	**2a**	0.08:2:200	60	83	15.2	12.0	1.27
7	**2a**	0.04:1:500	180	92	53.0	67.0	1.45
8	**2b**	0.08:2:100	200	95	7.0	6.9	1.26
9	**2b**	0.04:2:50	200	91	3.8	3.4	1.25
10	**2b**	0.04:2:100	200	89	7.2	6.5	1.23

^a^Polymerization conditions: *p*-MOS (1 equiv.), **1** (0.04 − 0.08 mol %), and **2a** or **2b** (1.0 − 4.0 mol %) at room temperature in CH_2_Cl_2_/Et_2_O (99/1 vol%) under green LED irradiation.

^b^Determined by GPC, relative to polystyrene standards. ^c^*M*_n_ (theo) is the theoretical number-average molar mass calculated on the basis of the equation *M*_n_ (theo) = [M]_0_/[CTA]_0_ × MW^M^ × conv.% +MW^CAT^, where [M]_0_, [CTA]_0_, MW^M^, and MW^CAT^ correspond to initial monomer concentration, initial CTA concentration, molecular weight of monomer, and molecular weight of CTA agent, respectively.

**Fig. 3:**
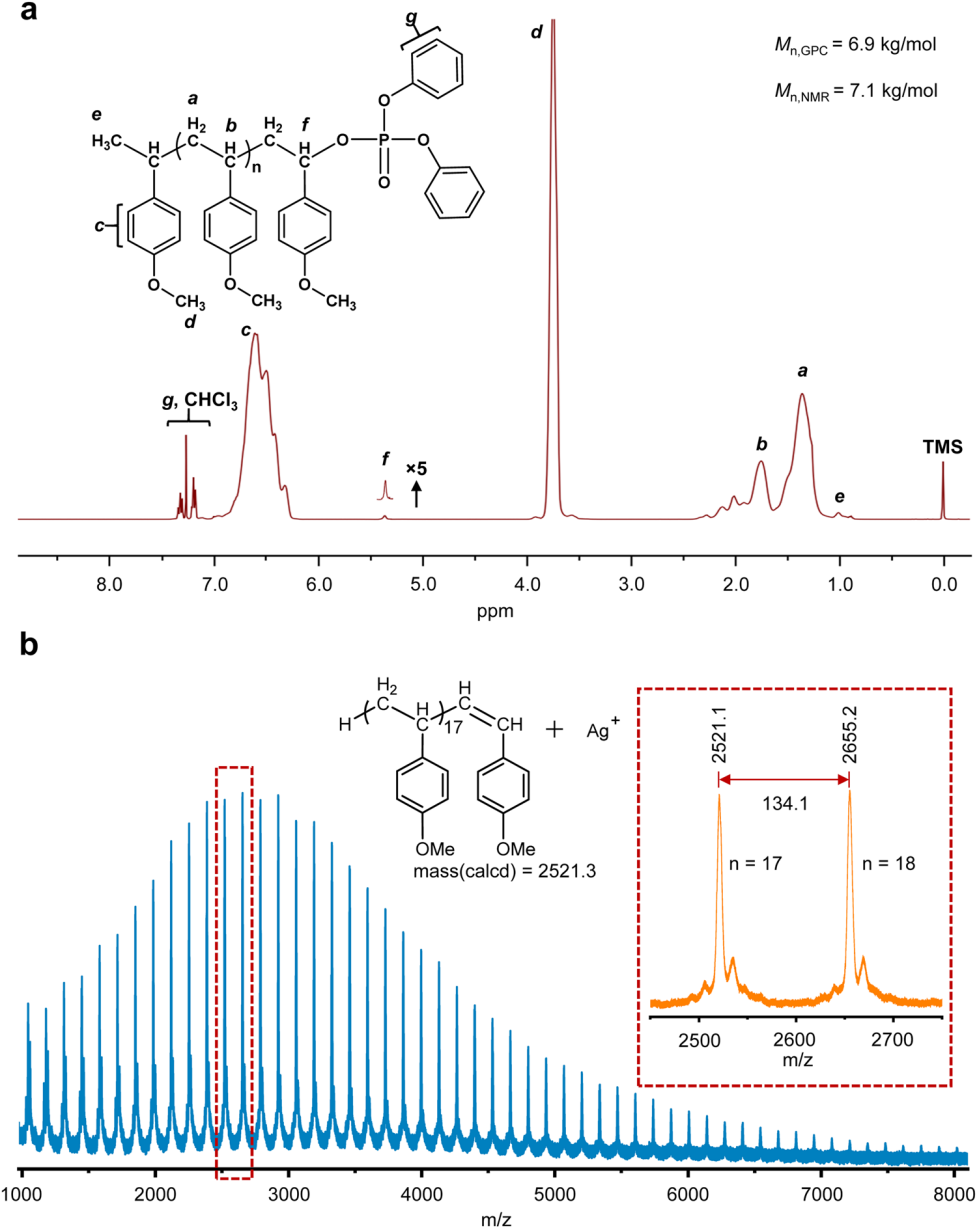
Characterizations of the polymers. **a** ^1^H NMR and **b** MALDI-TOF mass spectra of the resulting poly(*p*-MOS) with red box in the top right showing two peaks of 2521.1 and 2655.2 corresponding to poly(*p*-MOS) (*DP*_n_ =18 and 19) cationized by silver and bearing a terminal group of double bond.

The resulting polymers were characterized by ^1^H NMR spectroscopy. Taking the polymers from entry 3 for example (Fig. 3), observed were the prominent peaks assigned to the repeating units of poly(*p*-MOS) main-chain at 1.25–2.50 ppm (*a*, *b*), 3.79 ppm (*d*), and 6.60 ppm (*c*), respectively. In addition, the peaks of the chain ends were also found, with the small peaks at 7.20–7.42 ppm (*g*), 5.39 ppm (*f*) and 1.02 ppm (*e*) corresponding to the phenyl groups, the acetal-methine protons at the ω-chain end linked to the phosphate ester **2a**, and -C***H***_3_ in the α-chain end of the polymers, respectively. The number-average molar mass *M*_n_ of the polymers measured by gel permeation chromatography (GPC) is 6.9 kg/mol, which complies with the degree of polymerization (*DP*_n_ = H_d_/H_3f_, *M*_n, NMR_ = 7.1 kg/mol) calculated from the ^1^H NMR analysis.

The chemical nature of the resulting polymers was also examed by matrix-assisted laser desorption/ionization time-of-flight mass spectroscopy (MALDI-TOF MS). Due to the relatively weak P(O)O–R bonds of phosphate, the resulting poly(*p*-MOS) using **2a** as CTA bears dibutyl phosphate end group that is rather labile to undergo hydrolysis during the sample preparation process (Supplementary Fig. 17), a MALDI-TOF MS analysis of poly(*p*-MOS) using **2b** was therefore performed to further investigate the chain-end structure. It clearly confirms the silver cationized polymer chains bearing a methyl at one chain end, and the other end group is a double bond (Fig. 3b), which is commonly observed for poly(*p*-MOS) produced by cationic polymerization after strong laser bombarding during MALDI procedure^48^.

**Fig. 4:**
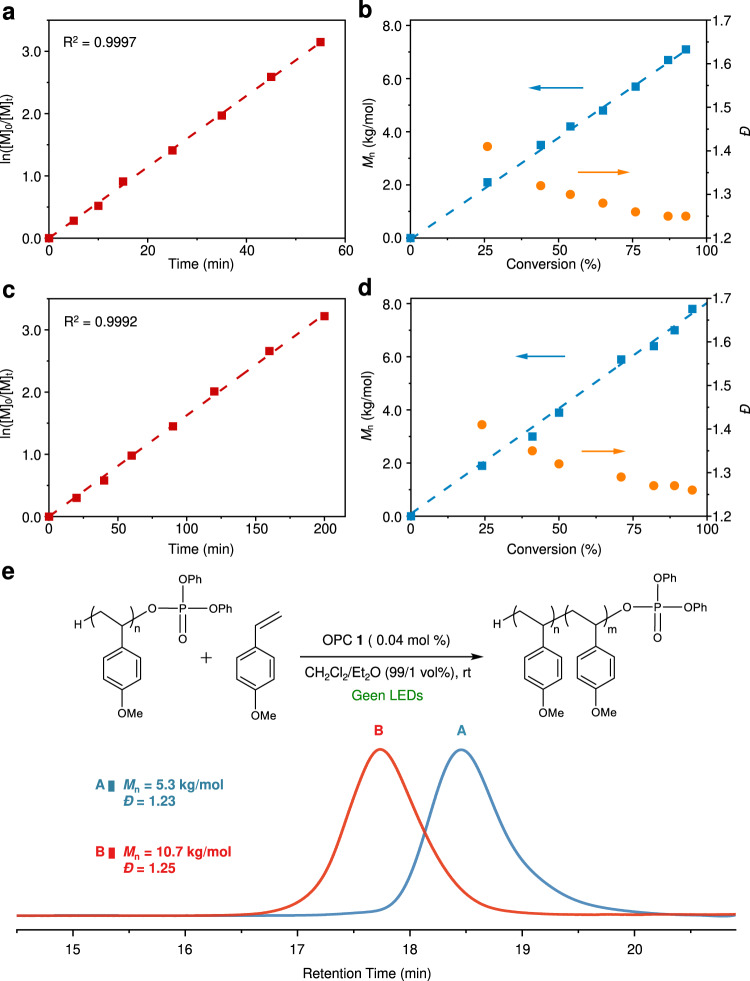
Kinetic study of the photo-induced polymerization. **a** and **c** plots of ln([M]_0_/[M]_t_) vs. reaction time (red squares) using **2a** and **2b** as CTA, respectively. **b** and **d** *M*_n_ (blue squares) and *Đ* (orange circles) of poly(*p*-MOS) using **2a** and **2b** as CTA, respectively. **e** GPC traces of sequential monomer addition experiment.

We also carried out the kinetic study of the photo-induced polymerization, which was monitored by ^1^H NMR and GPC. For both CTA **2a** and **2b**, the ln([M]_0_/[M]_t_) as a function of time (Fig. 4a, c) and plots of *M*_n_ against conversion (Fig. 4b, d) unambiguously reveal the first-order kinetic behavior, which therefore confirms the high degree of polymerization control. Besides, as expected for a living polymerization system, the molar mass dispersity (*Đ*) gradually decreased from 1.41 to 1.25 with the increase of monomer conversion (Fig. 4b, d). It is also noteworthy that **2a** accelerated the polymerization more than its dibutyl counterpart **2b** did, as the polymerization with **2a** was completed within 60 min (entries 3–6), while at least 200 min was needed (entries 8–10, Table 1). This tendency is in line with the greater bimolecular quenching constant *k*_q_ of **2a** during its PET process with OPC **1** (*vide supra*).

We further performed the sequential monomer addition experiment so as to verify the chain-end fidelity (Fig. 4e). After the full consumption of *p*-MOS that yielded the polymers with *M*_n_ = 5.3 kg/mol and *Đ* = 1.23, the irradiation was switched off. When another equivalent portion of monomer was added to the solution, the propagation resumed upon the turn-on of irradiation. This second step yielded the extended polymer with a *M*_n_ = 10.7 kg/mol, along with a monomodal distribution and low value of *Đ* = 1.25). The *M*_n_ increment of 5.4 kg/mol in the prolonged polymer chain agrees well with the theoretical value.

Taking together the first-order kinetic, predeterminable molar mass, low molar-mass distribution and chain-end fidelity, we therefore confirmed our photo-induced polymerization unambiguously exhibits a living feature. We then set out to search for the optimal reaction conditions (Table 1). We also found, increasing the catalyst loading raised the polymerization rate (entry 3 vs. entry 5). After optimizing the reaction conditions, we chose the parameters listed in entry 4, Table 1 for the following study.

### Excellent on-off photo-switching characteristics

**Fig. 5:**
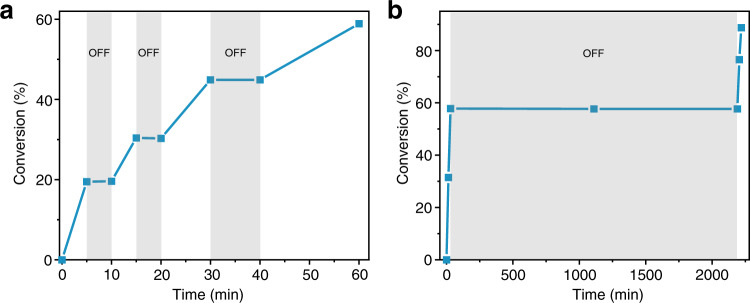
Temporal control of polymer chain growth via on-off light switching. **a** Monomer conversion of *p*-MOS by using OPC **1** and CTA **2a** with short dark intervals. **b** Prolonged dark period (36 h) to demonstrate excellent temporal control.

A unique feature of light-controlled polymerizations is their excellent temporal control over chain propagation by on-off switching of light source. We first attempted to stir the reaction mixture of *p*-MOS, **2a**, and **1** under the optimized conditions in dark for 12 h, and no polymerization was noticed. The intermittent on-off irradiations were then applied to the mixture, and ^1^H NMR was used to monitor at each switching point for the determination of conversion rate. The polymerization proceeded only under light irradiation, which immediately ceased upon removal of light stimulus (Fig. 5a). It is worth mentioning, after iteration of several on-off switching, that the final polymer exhibited a unimodal molar mass distribution with a low *Đ* value of 1.28, comparable to that of the as-synthesized polymers without intermittent exposure (Supplementary Fig. 19).

To better evaluate the photo-controlled characteristics of the living polymerization process, the intermittent irradiation experiments with a long interval in dark period of 24 or 36 h were respectively conducted, right after an initial photo-polymerization to a high rate of conversion was achieved (Fig. 5b). It was found that almost no change in monomer conversion occurred in these dark periods, as the reaction was totally halted; the reaction was woken again after the green light was turned on. This dormant period reached up to 36 h, which is, to the best of our knowledge, the longest reported so far for the photo-controlled cationic polymerization, indicating the considerable stability of OPC **1**, which therefore exerts excellent temporal control of the polymer propagation.

### Scope of photocatalysts, solvents and monomers

To investigate the influence of OPCs on this photo-controlled polymerization system, triphenylmethylium and tris(*p*-methoxyphenyl)methylium were used as photocatalysts to initiate the polymerization of *p*-MOS. For triphenylmethylium tetrafluoroborate, a rapid polymerization of *p*-MOS completed within several minutes without LED irradiation, yielding a 4.8 kg/mol poly(*p*-MOS) with a *Đ* of 1.68 (Supplementary Fig. 21). On the other hand, the polymerization proceeded in an uncontrolled fashion under photoirradiation.

When tris(*p*-methoxyphenyl)methylium was used as photocatalyst, polymerization of *p*-MOS did not occur without LED irradiation, while it proceeded readily under blue LED irradiation, yielding poly(*p*-MOS) with *M*_n_ of 8.1 kg/mol and *Đ* of 1.31 (Supplementary Fig. 22). However, the polymerization also proceeded during the off-period, indicating that tris(*p*-methoxyphenyl)methylium had poor photocontrol in this polymerization system (Supplementary Fig. 23). Thereby, triphenylmethylium and tris(*p*-methoxyphenyl)methylium are deemed unsuitable for photo-controlled cationic living polymerization of *p*-MOS.

We also studied the scope of the solvents for the polymerization. We first attempted the polymerization with nonpolar solvents including toluene, *n*-hexane, and diethyl ether, in which the OPC **1** couldn’t be well dissolved, and no conversion was observed after 4 h LED irradiation (Table 2, entries 1–3). On the other hand, polymerization in polar solvent, such as acetonitrile, produced only polymers with low molar mass (*M*_n_ = 3.7 kg/mol) with high dispersity (*Ð* = 2.13), suggesting that the polymerization process is unregulated (Table 2, entry 4).

**Table 2:** Details for polymerization with different solvents and monomers^a^.

Entry	monomer	solvent	Conv.%	*M*_n_^b^ (kg/mol)	*Đ*
1	*p*-MOS	toluene	0	–	–
2	*p*-MOS	*n*-hexane	0	–	–
3	*p*-MOS	Et_2_O	0	–	–
4^c^	*p*-MOS	acetonitrile	100	3.7	2.13
5	*p*-MOS	CH_2_Cl_2_:Et_2_O (90:10 vol%)	3	–	–
6	*p*-MOS	CH_2_Cl_2_:THF (90:10 vol%)	0	–	–
7	*p*-MOS	toluene:CH_2_Cl_2_:Et_2_O (50:49:1 vol%)	87	6.8	1.15
8	styrene	CH_2_Cl_2_:Et_2_O (99:1 vol%)	0	–	–
9	indene	CH_2_Cl_2_:Et_2_O (99:1 vol%)	0	–	–
10	4-methylphenylene	CH_2_Cl_2_:Et_2_O (99:1 vol%)	6	–	–
11	3,4-dimethoxystyrene	CH_2_Cl_2_:Et_2_O (99:1 vol%)	89	7.7	1.35
12^c^	*n*BVE	CH_2_Cl_2_:Et_2_O (99:1 vol%)	100	0.3	3.15
13	*n*BVE	*n*-hexane:CH_2_Cl_2_:Et_2_O (80:18:1 vol%)	100	4.9	1.25

^a^Polymerization conditions: [**1**]_0_: [**2a**]_0_: [monomer]_0_ = 0.04:1:50 at room temperature under green LED irradiation.

^b^Determined by GPC, relative to polystyrene standards.

^c^Reactions were initiated without light irradiation.

To further investigate the influence of the solvents, we tried to perform the photo-controlled polymerization of *p*-MOS in mixed solvent. When performed in a mixture of toluene, CH_2_Cl_2_, and Et_2_O (50: 49: 1 vol%), a poly(*p*-MOS) with *M*_n_ = 6.8 kg/mol and *Ð* = 1.15 was yielded after 3 h irradiation (Table 2, entry 7). It also showed that increasing the volume ratio of non-polar solvents may lower the molar mass dispersity of the resulting polymers (Table 2, entry 7 vs. entry 4).

We finally studied the polymerization with various monomers including styrene, indene, 4-methylphenylene, 3,4-dimethoxystyrene, and *n*-butyl vinyl ether (*n*BVE) (Table 2, entries 8–13) in the mixed solvents. Among the tested monomers only 3,4-dimethoxystyrene and *n*-butyl vinyl ether (*n*BVE) could yield the corresponding polymers. Particularly, the polymerization of *n*BVE in a mixture of *n*-hexane, CH_2_Cl_2_, and Et_2_O (80/18/2 vol%) yielded the polymer with predictable molar mass (*M*_n_ = 4.9 kg/mol) and low molar-mass dispersity (*Đ* = 1.25).

### The mechanistic study of photo-controlled living cationic polymerization

Since CTA **2a** or **2b** probably can be converted into cationic radical with OPC **1**^47^, the reaction reported herein can proceed either via radical or cationic polymerization. We first attempted in vain the polymerization of methyl acrylate (MA), which therefore presumably precludes the possibility of radical polymerization. Meanwhile, we found that the polymerization of *p*-MOS under the optimal conditions was quenched upon addition of the cationic scavenger MeOH (10 vol%), which therefore suggests the polymerization might comply with a cationic mechanism.

To get a further insight into the mechanism of photo-controlled living polymerization brought by phosphate CTA, the potentials corresponding to the onset of oxidation of CTA **2a**, **2b**, and *p*-MOS were determined in acetonitrile by cyclic voltammetry (Supplementary Fig. 24). It showed that the excited potential of **1** (E* = +1.55 V vs. SCE) was sufficient to oxidize both CTAs (E^0^_2a/2a•+_ = +0.72 V vs. SCE for **2a**, and E^0^_2b/2b•+_ = +0.69 V vs. SCE for **2b**) and *p*-MOS (E^0^ = +1.09 V vs. SCE). As mentioned above, although the monomer can be oxidized by the photo-excited OPC **1**, the polymerization proceeded in an uncontrolled manner. On the other hand, excellent control was achieved in the presence of CTA. Together with these polymerization experiments in comparison, the different potentials reveal that, as compared with *p*-MOS, **2a** and **2b** are more readily to be oxidized by OPC **1**. For CTA **2a**, both the Stern-Volmer plot (Supplementary Fig. 13) and the oxidation potential indicate PET process between **2a** and OPC **1** dominates the oxidation process. For CTA **2b**, the faster fluorescence quenching of *p*-MOS vs. CTA **2b** (Supplementary Fig. 13) would suggest the electron transfer process between OPC **1** and CTA **2b** might be the rate-determining step. It therefore implies that once the oxidized monomers are formed, if there is any, may undergo a second electron transfer to the CTA molecules, which prevents the uncontrolled polymerization by direct oxidation of monomers with OPC **1** (Table 1, Entry 2)^43^.

We also conducted the electro-polymerization of *p*-MOS with CTA **2b** for reference. The onset of oxidation potential of the **2b** and *p*-MOS mixture was determined to be +0.72 V vs. SCE (Supplementary Fig. 25), which is 0.37 V lower than that of pure *p*-MOS. At the potential of +0.72 V, this mixture was found to yield the polymers with high dispersity (*M*_n_ = 8.9 kg/mol, *Đ* =1.87, Supplementary Fig. 26), whereas no polymerization was noticed in the absence of **2b** under the same conditions. This result confirms the hypothesis that the major pathway for this light-controlled polymerization proceeds via direct oxidation of phosphate CTA.

**Fig. 6:**
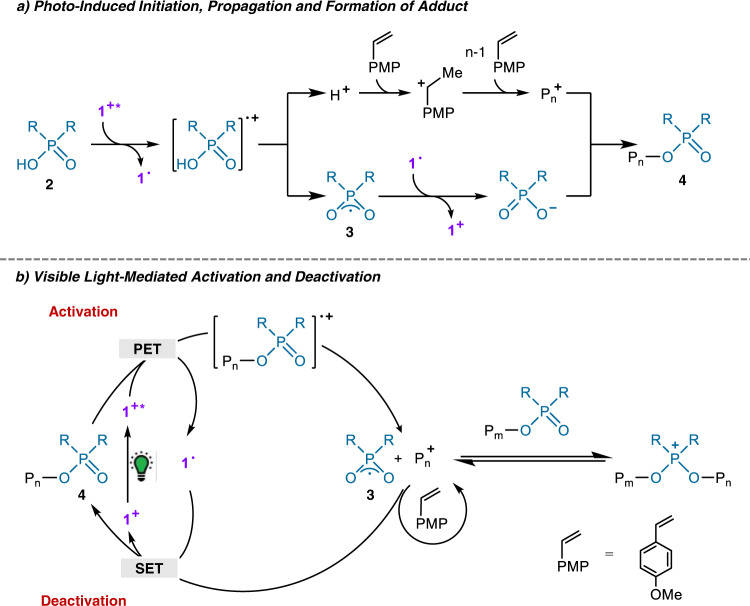
Proposed mechanism for the photo-controlled living cationic polymerization of *p*-MOS with OPC 1 and phosphate CTA. **a** Photo-induced initiation, propagation and formation of adduct. **b** Visible light-mediated activation and deactivation.

Putting all the evidences together, we therefore propose that the photo-controlled living cationic polymerization proceeds as follows (Fig. 6). Upon photo irradiation under green LED, the cation of OPC **1** is excited, which oxidizes the phosphate CTA **2** via PET process, leading to the formation of the corresponding radical cation (Fig. 6). The subsequent mesolytic cleavage of this radical cation generates two species: firstly, it releases a proton to initiate *p*-MOS monomer and creates an active cationic species, which then induces the chain propagation, presented as P_n_^⊕^; secondly, it also generates species **3**, which is reduced by radical **1•** to yield the phosphate anion. The resulting P_n_^⊕^ and phosphate anions could combine into an adduct **4** as the dormant species.

Subsequently, the phosphate of **4** can be constantly oxidized by the excited OPC **1** and keeps releasing the active cation for polymer chain growth (Fig. 6)^49^. With the presence of phosphate **4**, the chain propagation is controlled via a phosphonium intermediate that participates in a RAFT-like degenerative chain transfer process. Ultimately, the cationic species P_n_^⊕^ and radicals **3** can be deactivated by the SET process from **1•** to regenerate the dormant species **4**. The activation and deactivation process happen continuously, leading to the iterative polymer chain growth thorough a living cationic polymerization process. However, when the light turns off, the catalytic cycle will be closed at the species **4**, which can be reactivated when the light turns on.

### Decoloration of visible light-controlled polymerization

**Fig. 7:**
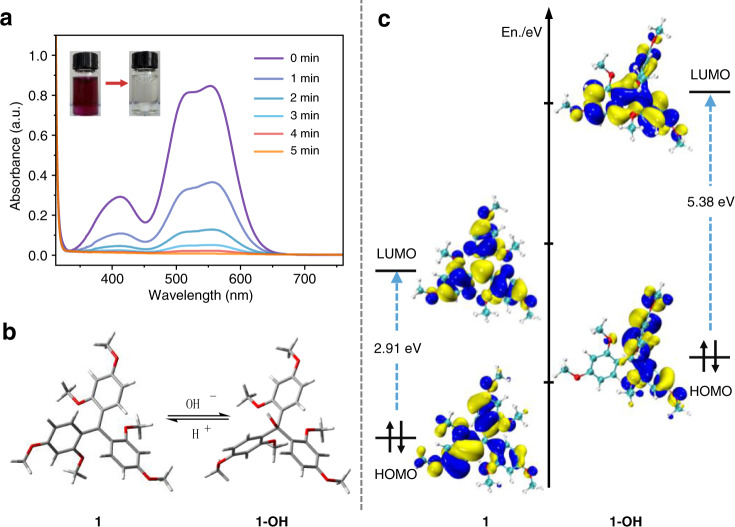
Decoloration of the OPC 1 and proposed mechanism of decoloration. **a** UV/vis absorption spectrum of the polymerization mixture after addition of triethylamine, inset showing the corresponding apparent color change of the mixture solution. **b** Optimized chemical conformation of OPC **1** and **1-OH** and their transition mechanism (DFT, B3LYP/6–31 G*). **c** Frontier orbitals, energy levels and their potential gaps of the ground state of OPC **1** (left) and **1-OH** (right) calculated by DFT (B3LYP/6–31 G*).

As stated in the Introduction, it is nontrivial and costly to remove residual photocatalysts of photoinduced polymerization, which impose color contamination^14^ and polymer degradation^37^ of the resulting polymers. By virtue of the Lewis acid-nature of our OPC **1**, we reasoned that it could be simultaneously decolored and deactivated by simple addition of a base after the reaction. We therefore set out to verify our assumption by adding 2-fold molar excess of triethylamine relative to OPC **1** into the reaction mixture. To our delight, the erstwhile purple color of the solution started fading until this total disappearance within five minutes (Fig. 7a, inset), and this color change was also witnessed by UV/vis spectroscopy (Fig. 7a).

To understand the decoloration mechanism, the density functional theory (DFT, B3LYP/6–31 G*) calculation of **1** and its leuco-hydroxide derivative (**1-OH**) was performed. As shown in Fig. 7b, the three-blade propeller structure of OPC **1** with *D*_3_ symmetry in its cationic form was obtained after the geometry optimization, which is composed of three aryl rings linked to a *sp*^2^-hybridized central carbon atom, and the three bond-angles are all 119.9°. Due to the extensive delocalization of electrons on the arenes at its HOMO, it exhibits an energy gap of 2.91 eV within its LUMO. It therefore indicates the cation **1** is a strong visible-light absorbent, which should absorb green light^20^. Whereas the configuration of neutralized form **1-OH** is dramatically different, as the bond angles between three C-C bonds connected to *sp*^3^-hybridized central carbon atom are 114.7°, 117.7°, and 107.8°, respectively. It reveals that the configuration of **1-OH** is similar to a tetrahedral configuration. Unlike electrons delocalized over a wider range in the HOMO of the cationic form, the electrons of leuco-hydroxide **1-OH** are delocalized within a smaller range, resulting in a much larger energy gap (5.38 eV) between its HOMO and LUMO, revealing no absorption in the visible range at all. (Fig. 7). This DFT calculation and comparision therefore explains the color change from purple cation **1** to its colorless neutralized form **1-OH**.

To verify that the leuco-hydroxide derivative of OPC **1** indeed forfeits its inherent photocatalytic activity after its visible absorption vanishes, we attempted the photo-induced cationic polymerization by using **1-OH** as the OPC. No polymerization product was observed, indicating that the leuco-hydroxide derivative of OPC **1** is photocatalytic inactive, and the quenching of the residual OPC **1** is easily achieved by simple addition of a base, concomitant with its decoloration.

## Discussion

The photo-controlled living cationic polymerization of *p*-MOS using a metal-free organo-photocatalyst **1** and phosphate CTA **2** is showcased in the current manuscript. This living cationic polymerization not only successfully yielded poly(*p*-MOS) with predetermined molar mass and low *Đ*, but also exhibits excellent stable photo-control characteristics, which can inhibit chain growth during long dark periods (>36 h). Most notably, we demonstrated, for the first time, that the erstwhile dark purple reaction mixture was easily decolored by simple addition of a base, yielding the resulting polymers as white powders. This is due to the deactivation of the OPC **1** cation, forming its **1-OH** neutral form, which will no longer impose potential photolysis to the polymer product. Furthermore, the phosphate substituents can be readily tuned, so that different types of polymers are envisioned, which are in progress in our research group and will be reported in due course.

## Methods

### Procedure for photo-controlled cationic polymerization of *p*-MOS

In an argon-filled glovebox, *p*-MOS (0.67 mL, 5.0 mmol, 50.0 equiv), 0.2 mL of a stock solution of phosphate CTA (**2**) in DCM (0.5 mM, 0.1 mmol, 1.0 equiv), 0.2 mL of a stock solution of OPC **1** in DCM (0.02 mM, 0.004 mmol, 0.04 equiv), and 0.4 mL mixed solvent CH_2_Cl_2_/Et_2_O (98/2 vol%) were charged in an oven-dried one-dram vial with a stir bar. A septum cover was then applied to the vial, which was then put outside the glove box in front of a green LEDs spot lamp (5 W, *λ*_max_ = 532 nm, 30 mW/cm^2^). Generally, full conversion was reached within 1–4 h. The solvent was then removed from the polymerization reaction mixture by rotatory evaporation under vacuum, yielding the pure polymer. For details, additional data, and experiments, please see the Supplementary Information (Materials, Supplementary Methods, Supplementary Figs. 1–31, etc.).

## Supplementary information

Supplementary information

## Data Availability

The authors declare that all data supporting the results of this work, including those in this paper or in the Supplementary Information, are available. Additionally, data can be provided upon request from the corresponding author.
