# Supplementary material for: Visible light-controlled living cationic polymerization of methoxystyrene
Source: Nat Commun. 2022 Jun 24;13:3621. doi: 10.1038/s41467-022-31359-4 (PMC9232534; doi:10.1038/s41467-022-31359-4)
Supplement: Supplementary file 1 — Supplementary information [file 41467_2022_31359_MOESM1_ESM.pdf]

Supplementary Information for

Visible Light-Controlled Living Cationic Polymerization of Methoxystyrene

Lei Wang[1], Yupo Xu[1], Quan Zuo[1], Haojie Dai[1], Lei Huang[1], Meng Zhang[1], Yongli Zheng[1], Chunyang Yu[1], Shaodong Zhang[1]*, Yongfeng Zhou[1]*

[1]School of Chemistry and Chemical Engineering, State Key Laboratory of Metal Matrix Composites, Shanghai Jiao Tong University, 800 Dongchuan Road, Shanghai 200240, China

Table of Contents

1. Experimental Section

2. Supplementary Figures 1-31

3. Supplementary Table 1

4. Supplementary References

General Reagent Information

Tris(2,4-dimethoxyphenyl) methanol (98%), tetrafluoroboric acid-diethyl ether complex (50-55%), diphenyl phosphate (99%), dibutyl phosphate (97%), tetra-*n*-butylammonium hexafluorophosphate (99%) were purchased from Adamas-beta. 4-Methoxystyrene (96%) was purchased from Aladdin and distilled twice over calcium hydride (CaH_2) under reduced pressure and stored under argon atmosphere at -20°C before use. Dichloromethane (DCM, HPLC), acetonitrile (ACN, HPLC), methanol (MeOH, HPLC), and diethyl ether (Et_2O, ≥99.5%) were purchased from Adamas and stored in Schlenk bar under argon atmosphere followed by being transferred into the glovebox.

General Analytical Information

^{1}H NMR， ^{13}C NMR and ^{31}P NMR spectra were recorded using Bruker AVANCEIII HD 400 MHz or Bruker AVANCE III HD 500 MHz spectrometer with $CDCl_3$ as solvents at 298K. Tetramethylsilane (TMS) was used as an internal standard. GPC measurements were carried out using an HLC-8320GPC (TOSOH, EcoSEC GPC System) system at 40 ^{o}C with THF as mobile phase at a flow rate of 1.0 mL/min. All number-average molecular weights (M_n), weight-average molecular weights (M_w), and dispersities ($Đ$) were determined relative to polystyrene standards. The UV-vis absorption spectra of sample were recorded at 298K in the range of 200-800 nm on a Shimadzu UV 3600 spectrometer. The fluorescence quenching spectra were recorded on a PTI QM/TM/IM steady-state & time-resolved fluorescence spectro-fluorometer (USA/CAN Photon Technology International Int.). ESR spectra were recorded on a Bruker EMX plus-9.5/12 ESR spectrometer. MALDI-TOF-MS was performed on a solariX XR 7.0 T hybrid quadrupole-FTICR mass spectrometer equipped with an ESI/APCI/MALDI ion source (Bruker Daltonics, Bremen,Germany).

General Photoreaction Setup

All manipulations for the photo-controlled cationic polymerization were set up in an

Argon atmosphere glove-box. The reactions were conducted under a 5 W green diode led® SEEWEE lights, and the distance between the one-dram vial and LED beads is ca. 4 cm. The light intensity at the place of vessel was recorded at 30 mW/cm^2. The emission spectra of the green LEDs were recorded on a Perfect Light PL-MW 2000 photoradiometer.

Synthesis of tris(2,4-dimethoxyphenyl)methylium tetrafluoroborate (1)

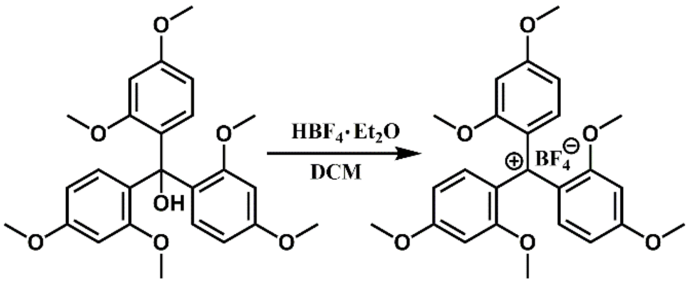

In a flame dried flask, a solution of HBF_4 in Et_2O (0.52 mL, 2.0 M, 1.0 mmol, 1.2 equiv) was added dropwise to a solution of tris(2,4-dimethoxyphenyl) methanol (352 mg, 0.88 mmol, 1.0 equiv) in DCM (5 mL) over 5 minutes at 0 °C and stirred for 1.5 hours under nitrogen. This solution was then added dropwise in Et_2O (50 mL) over 5 min at 0 °C, followed by filtration and washing with Et_2O, and finally drying under vacuum at room temperature. To purify the product further, it was dissolved in a small volume of DCM and reprecipitated in excess dry cold Et_2O, filtered and dried under vacuum to yield a dark purple powder (399 mg, 83% yield).

Tris(2,4-dimethoxyphenyl)methylium tetrafluoroborate (**1**): ^{1}H NMR ($CDCl_3$, 400 MHz) δ 7.05 (d, 3 H), 6.62-6.58 (m, 6 H), 4.07 (s, 9 H), 3.76 (s, 9 H) ppm； ^{13}C NMR ($CDCl_3$, 100 MHz): δ 180.26, 171.02, 165.47, 140.93, 124.67, 108.70, 97.956, 5.79, 56.40； UV/Vis: λ_{max} 553 nm; HRMS (m/z): $[M]^+$ calcd. for C25H27O6, 423.18; found, 423.1794.

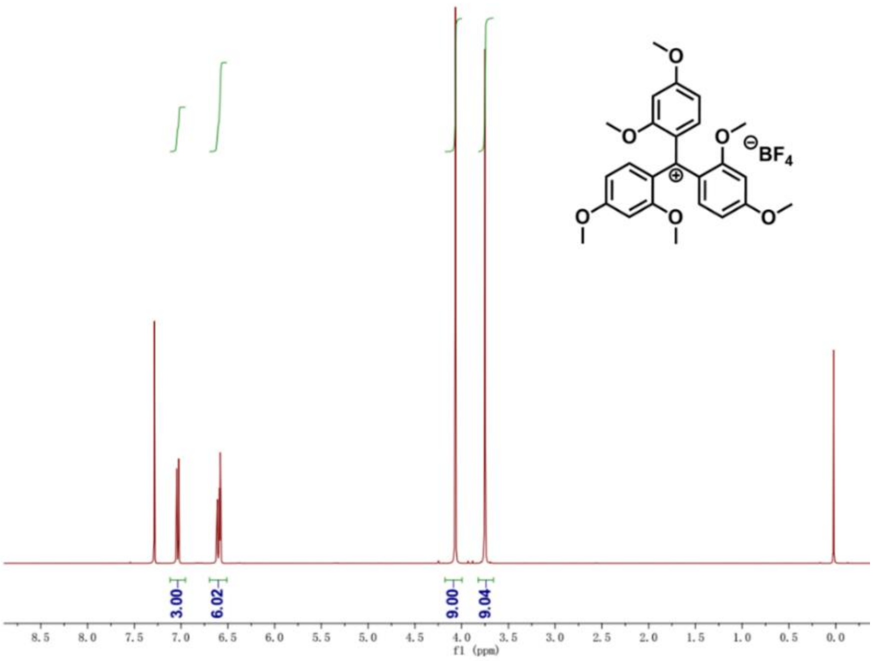

Supplementary Fig. 1. ^{1}H NMR of tris(4-dimethoxyphenyl)methylium tetrafluoroborate (**1**).

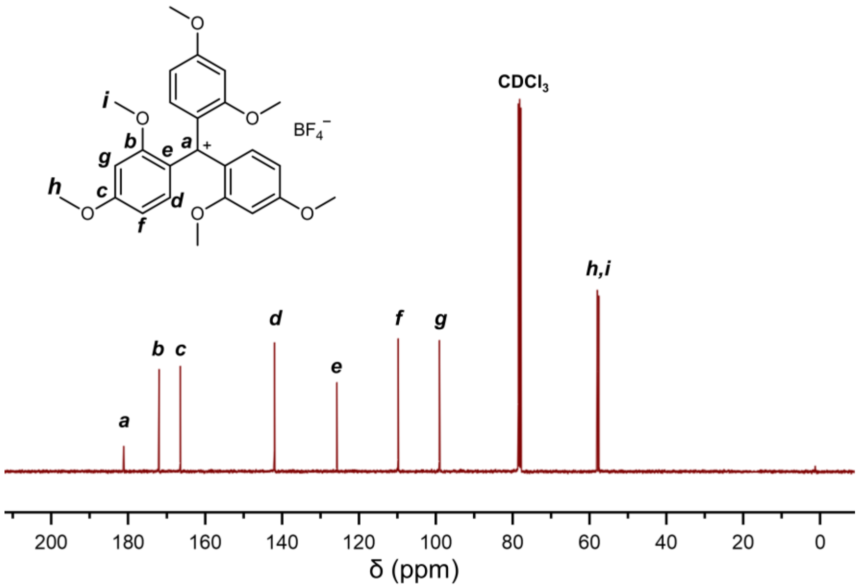

Supplementary Fig. 2. ^{13}C NMR of tris(4-dimethoxyphenyl)methylium tetrafluoroborate (**1**).

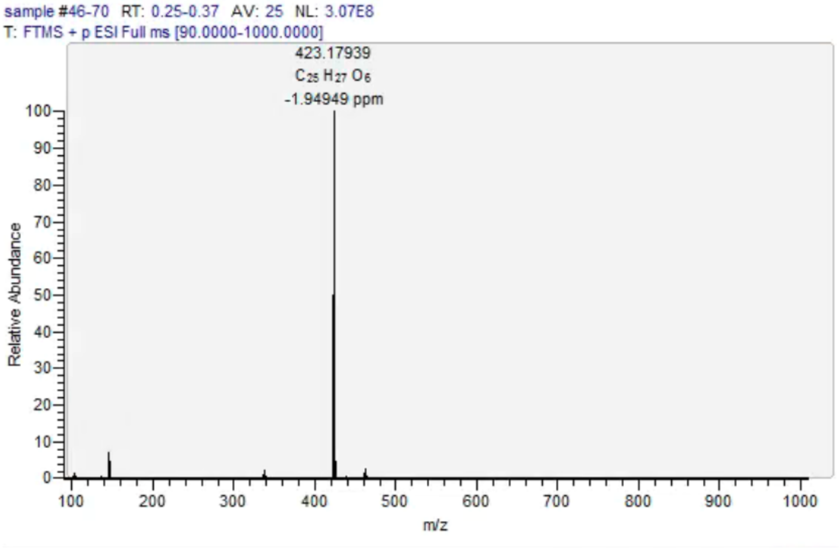

Supplementary Fig. 3. Mass spectra of tris(4-dimethoxyphenyl)methylium tetrafluoroborate (**1**).

Procedure for Photo-controlled Cationic Polymerization of 4-Methoxystyrene

In an argon-filled glovebox, 4-methoxystyrene (0.67 mL, 5.0 mmol, 50.0 equiv), 0.2 mL of a stock solution of phosphate CTA (**2**) in DCM (0.5mM, 0.1 mmol, 1.0 equiv), 0.2 mL of a stock solution of OPC **1** in DCM (0.02mM, 0.004 mmol, 0.04 equiv), and 0.4 mL mixed solvent CH_2Cl_2/Et_2O (98/2 vol%) were charged in an oven-dried one-dram vial with a stir bar. A septum cover was then applied to the vial, which was then put outside the glove box in front of a Green LEDs spot lamp (5 W, λ_{max} = 532 nm, 30 mW/cm^2). Generally, full conversion was reached within 1–4 hours. The solvent was then removed from the polymerization reaction mixture by rotatory evaporation under vacuum, yielding the pure polymer. The ^{1}H NMR for poly(4-methoxystyrene) using **2a** as CTA is shown in Figure 3a. The ^{1}H NMR for poly(4-methoxystyrene) using **2b** as CTA is shown in Figure S2. The ^{1}H NMR analysis for poly(4-methoxystyrene) (**2a** as CTA) precipitated with methanol is shown in Figure S4. The spectrum shows a small peak assigned to the methoxy (*h*) protons at the ω-chain end, indicating that the dormant phosphoric acid ester bond was substituted by methanol.

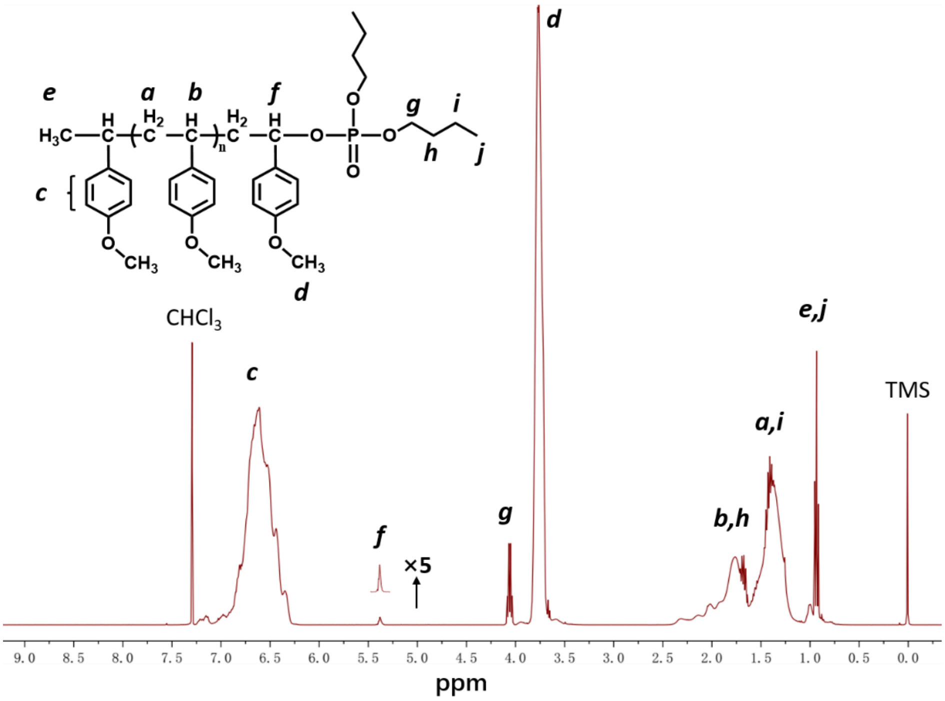

Supplementary Fig. 4. ^{1}H NMR spectra of poly(4-methoxystyrene) using **2b** as CTA; M_n = 7.0 kg/mol, $Đ$ = 1.26.

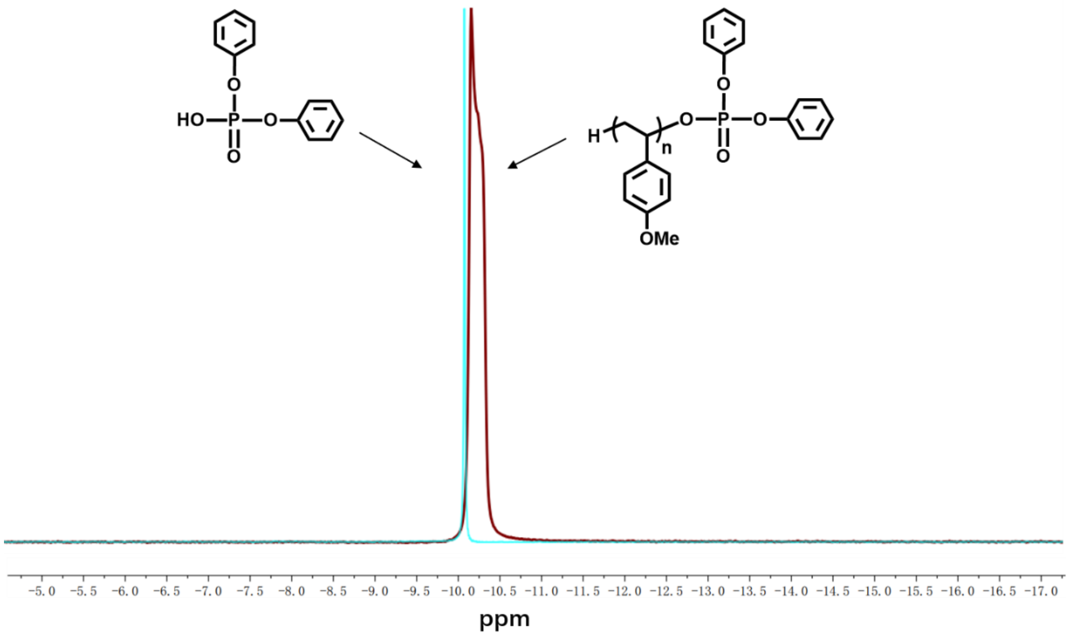

Supplementary Fig. 5. ^{31}P NMR spectra of poly(4-methoxystyrene) using **2a** as CTA (red) and **2a** (cyan).

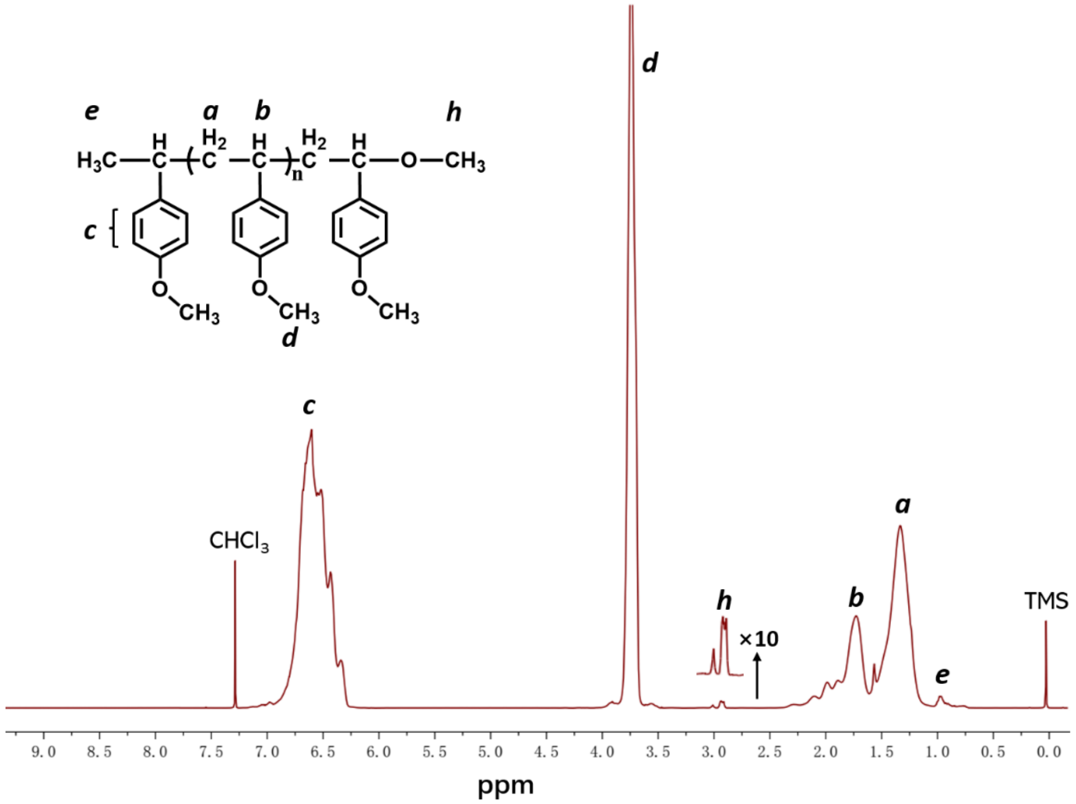

Supplementary Fig. 6. ^{1}H NMR of poly(4-methoxystyrene) precipitated with methanol; M_n = 7.6 kg/mol, $Đ$ = 1.28.

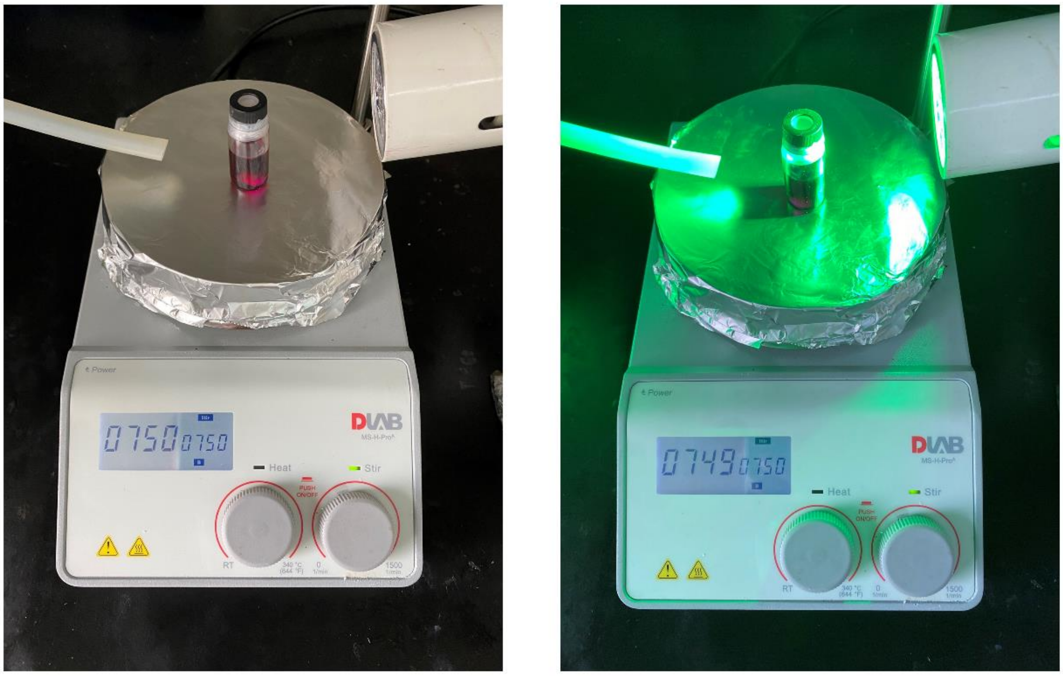

Supplementary Fig. 7. Photo polymerization reaction setup.

Procedure for Ultraviolet-visible Absorption Spectroscopy.

The UV-vis absorption spectra of sample were recorded at 298K in the range of 200-800 nm on a Shimadzu UV 3600 spectrometer. The solutions of the samples were added to a 1 cm quartz cuvette with sealed plug in an argon-filled glove box for the measurements under argon atmosphere.

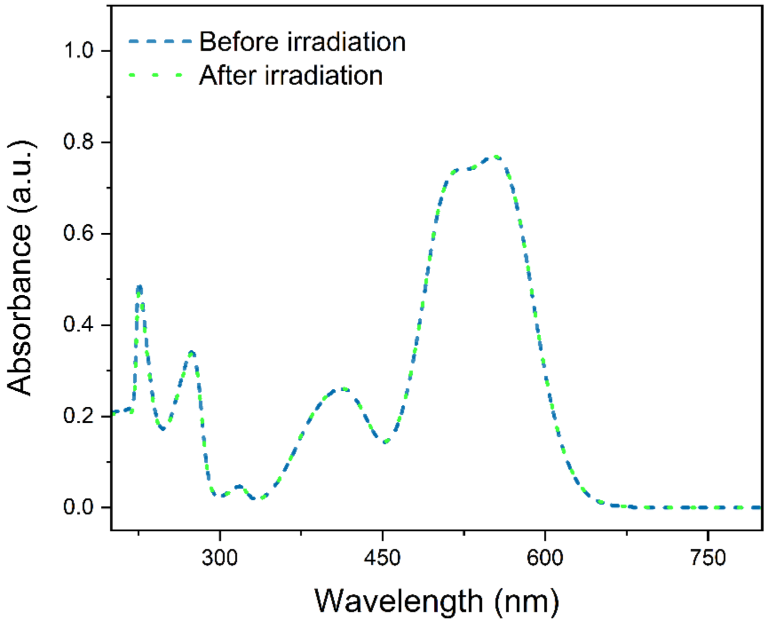

Supplementary Fig. 8. UV−visible spectra of 1.4×10^{-5} M OPC **1** in DCM before and after 12 h 5 W green LED irradiation.

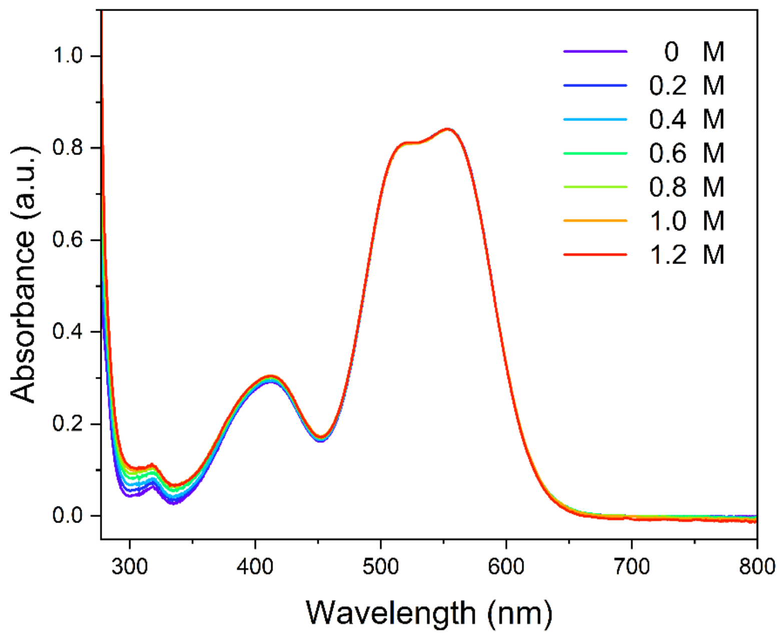

Supplementary Fig. 9. UV−visible spectra of 1.5×10^{-5} M OPC **1** in DCM with various concentrations of **2a**.

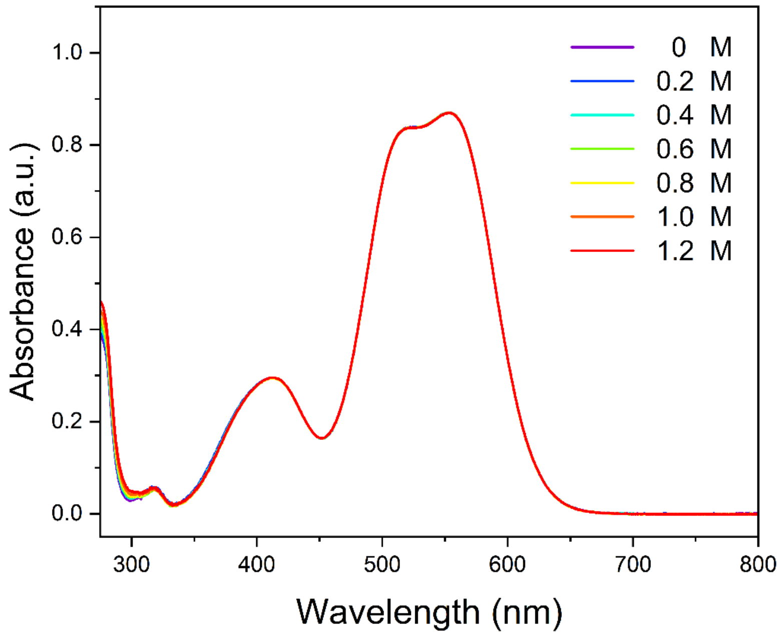

Supplementary Fig. 10. UV−visible spectra of 1.5×10^{-5} M OPC **1** in DCM with various concentrations of **2b**.

Procedure for Fluorescence Quenching Studies

The fluorescence quenching spectra were recorded on a PTI QM/TM/IM steady-state & time-resolved fluorescence spectro-fluorometer (USA/CAN Photon Technology International Int.) spectrometer. Step increment was set as 1 nm, and scan speed was set as 600 nm/min.

The solutions of OPC **1** (0.02 mM) in DCM were prepared in an argon-filled glove box and sealed before being taken out of the box. The fluorescence spectra were excited at 532 nm and recorded between 550 and 820 nm at various concentrations of **2a** (0 – 0.06 M). A concentration dependent fluorescence quenching was observed, as shown in Figures 2c. The emissions of OPC **1** (0.02 mM) at higher concentrations of **2b** (0 – 1.5 M) were also measured. A concentration dependent fluorescence quenching was observed at molar concentrations, as shown in Figures S8.

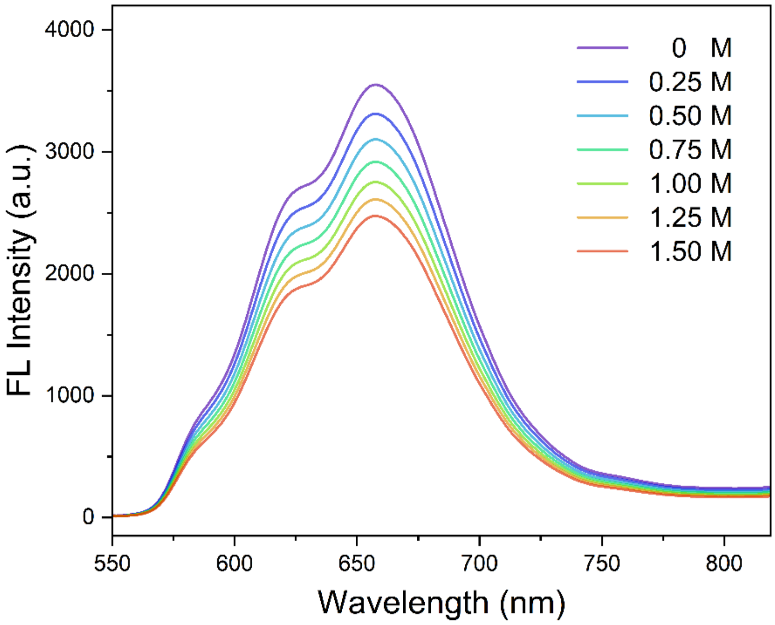

Supplementary Fig. 11. Spectral changes of the photo-catalyst **1** fluorescence with the addition of **2b**.

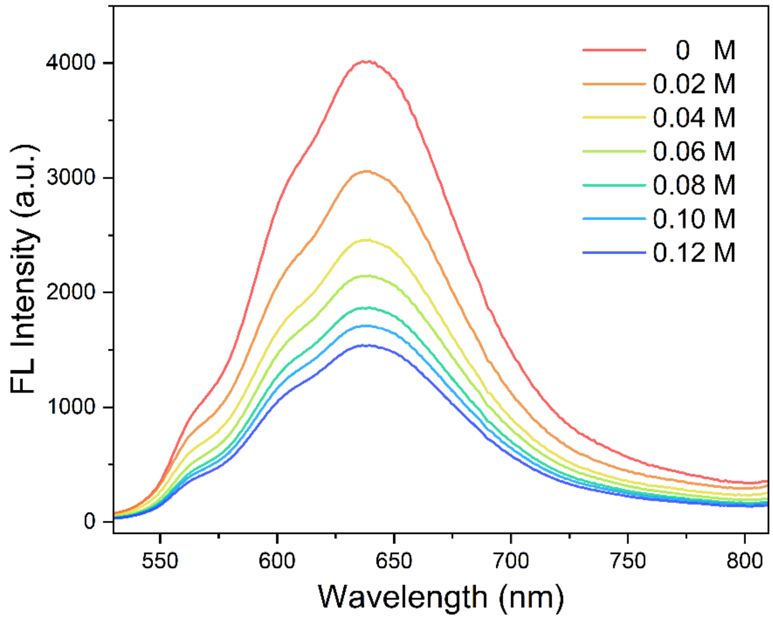

Supplementary Fig. 12. Fluorescence changes of the photocatalyst **1** with the addition of *p*-MOS.

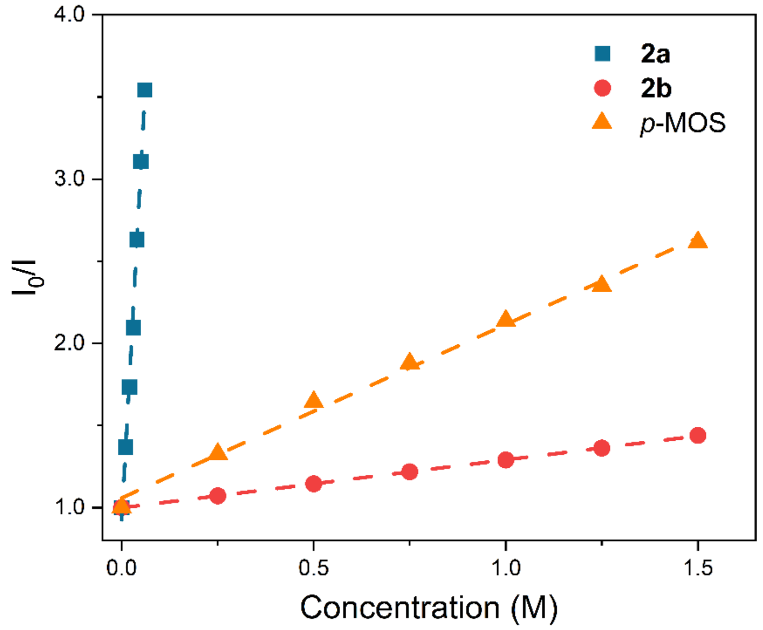

Supplementary Fig. 13. Stern-Volmer plots for the fluorescence quenching of OPC **1** by **2a**, **2b** and *p*-MOS.

Procedure for Time-Resolved Fluorescence Quenching Experiments

Time-resolved fluorescence spectra measurements were performed using PTI QM/TM/IM steady-state & time-resolved fluorescence spectro-fluorometer (USA/CAN Photon Technology International Int.). The solutions of OPC **1** (0.02 mM) at various concentrations of **2a** (0 – 0.06 M) in DCM were prepared in an argon-filled glove box and sealed before being taken out of the box. The fluorescence decay curves of samples were excited using 532 nm pulsed excitation and recorded, which shows the lifetime of OPC **1** continuously decreased with increasing amount of **2a** (Figure S10, τ_0 = 2.91 ns).

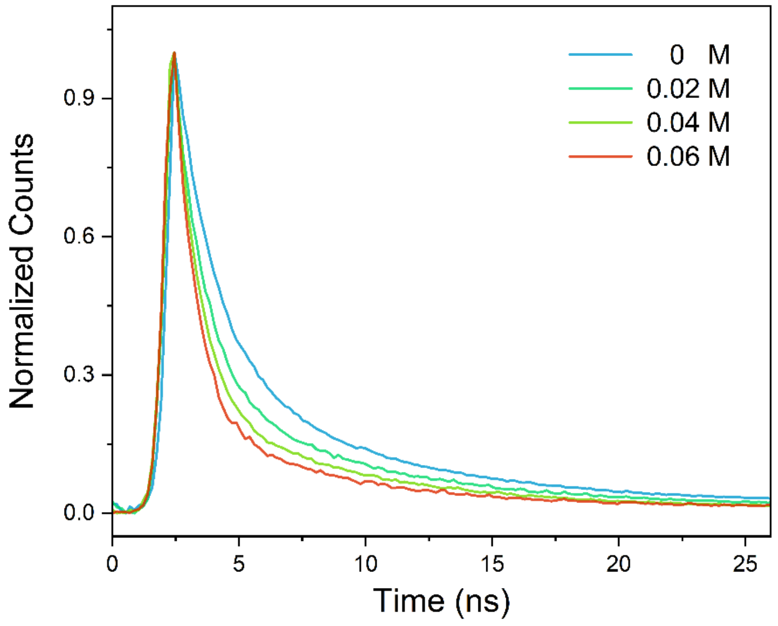

Supplementary Fig. 14. Fluorescence decay of 1×10^{-5} M **1** in DCM after a 532 nm pulsed excitation at varying concentrations of CTA **2a**.

Procedure for Electron Spin Resonance Spectroscopy.

ESR spectra were recorded on a Bruker EMX plus-9.5/12 ESR spectrometer. The acquisition parameters were fixed to 10 mW microwave power, 30 dB receiver gain, and 100 kHz modulation frequency. All samples were prepared in an argon-filled glove box and sealed before being taken out of the box. Samples in MeCN (20 μl) were transferred into quartz capillaries of 1.0 mm i.d. and 2.0 mm o.d. capillaries were then centered in standard 4 mm o.d. ESR quartz tubes. The solution of OPC **1** (0.1 M) or **2a** (0.1 M) in acetonitrile were irradiated (532 nm, 60W luminous power) and monitored by ESR with no obvious signal after 60 scans accumulating (Figure S11). A mixture solution of **1** (0.1 M) and **2a** (0.1 M) shows no signal in the absence of irradiation, while an ESR signal with g = 2.004, corresponding to a free radical species that can be assigned to 1• generated under steady-state irradiation (532 nm, 60 W luminous power) after a 60 scans accumulation (Figure 2d).

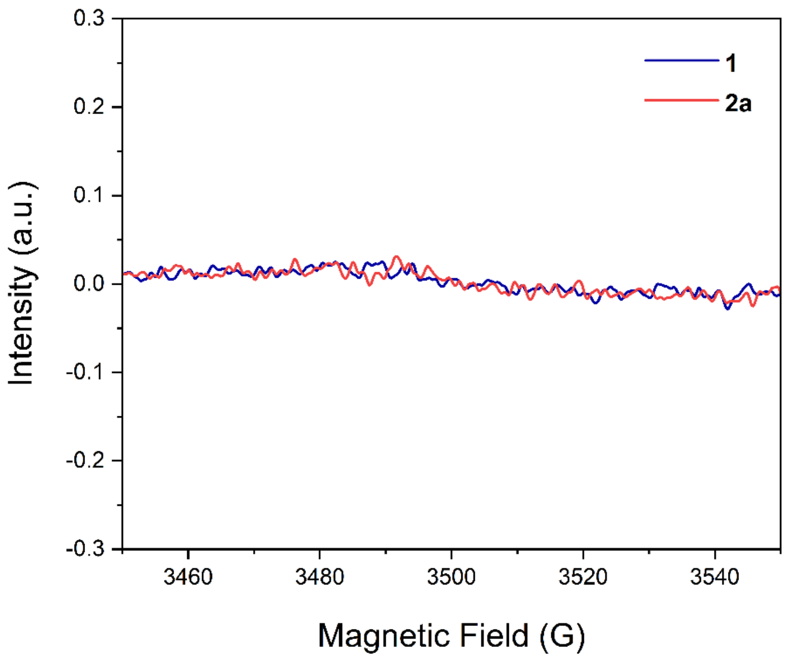

Supplementary Fig. 15. Electron spin resonance spectrum of 0.1 M **1** or 0.1 M **2a** in acetonitrile under steady-state 60 W 532 nm irradiation.

Procedure for Chain Extension Experiments

In an argon-filled glovebox, 4-methoxystyrene (0.40 mL, 3.0 mmol, 30.0 equiv), 0.2 mL of a stock solution of phosphate CTA (**2**) in DCM (0.5 mM, 0.1 mmol, 1.0 equiv), 0.2 mL of a stock solution of OPC **1** in DCM (0.02 mM, 0.004 mmol, 0.04 equiv), and 0.4 mL mixed solvent CH_2Cl_2/Et_2O (98/2 vol%) were charged in an oven-dried one-dram vial with a stir bar. A septum cover was then applied to the vial, which was then put outside the glove box in front of a Green LEDs spot lamp (5 W, λ_{max} = 532 nm, 30 mW/cm^2). The reaction was run to full conversion (1 hour). Then the vial was brought back in the glove box and an aliquot for ^{1}H NMR and GPC analysis (Conv. = 97%, M_n = 5.3 kg/mol, $Đ$ = 1.23) was taken prior to the addition of 4-methoxystyrene (0.40 mL, 3.0 mmol, 30.0 equiv). Then placed the vial next to the Green LEDs spot lamp again, and stirred while cooling by blowing compressed air over the reaction vial until the reaction reached full conversion (1 hour). The solvent was removed under vacuo to yield the pure polymer. The ^{1}H NMR and GPC analysis (Conv. = 93%, M_n = 10.7 kg/mol, $Đ$ = 1.25) for chain extension experiment is shown in Figure 4e.

Procedure for Kinetic Investigation of Photo-controlled Living Cationic Polymerization of 4-Methoxystyrene with OPC 1 as the Photocatalyst and 2a or 2b as the CTA

In an argon-filled glovebox, 4-methoxystyrene (0.67 mL, 5.0 mmol, 50.0 equiv.), 0.2 mL of a stock solution of phosphate CTA (**2**) in DCM (0.5 mM, 0.1 mmol, 1.0 equiv.), 0.2 mL of a stock solution of OPC **1** in DCM (0.02 mM, 0.004 mmol, 0.04 equiv.), and 0.4 mL mixed solvent CH_2Cl_2/Et_2O (98/2 vol%) were charged in an oven-dried one-dram vial with a stir bar. A septum cover was then applied to the vial, which was then put outside the glove box in front of a Green LEDs spot lamp (5 W, λ_{max} = 532 nm, 30 mW/cm^2). Aliquots were taken after 5 min, 10 min, 15 min, 25 min, 35 min, 45 min, and 55 min under positive argon pressure and subjected to ^{1}H NMR and GPC analysis when using **2a** as CTA (Figure 4a). Aliquots were taken after 20 min, 40 min, 60 min, 90 min, 120 min, 160 min, and 200 min under positive argon pressure and subjected to ^{1}H NMR and GPC analysis when using **2b** as CTA (Figure 4b).

Matrix-assisted Laser Desorption and Ionization Time-of-flight Mass Spectrometry (MALDITOF-MS)

MALDI-TOF-MS was performed on a solariX XR 7.0 T hybrid quadrupole-FTICR mass spectrometer equipped with an ESI/APCI/MALDI ion source (Bruker Daltonics, Bremen,Germany). The MS instrument was tuned and calibrated with ESI-L low concentration tuning mix (Agilent Technologies, Santa Clara, CA, USA) and sodium formate. The analytical sample was prepared by mixing the polymer solution (5.0 mg/mL in DCM) with the matrix solution (10 mg/mL DCTB in DCM) and silver nitrate (0.01 M in H_2O) in a volume ratio of 5/25/1, and then loaded onto the MALDI plate. The sample on the plate was thoroughly dried prior to analysis. The mass spectra were analyzed using Compass Data Analysis 5.0 (Bruker).

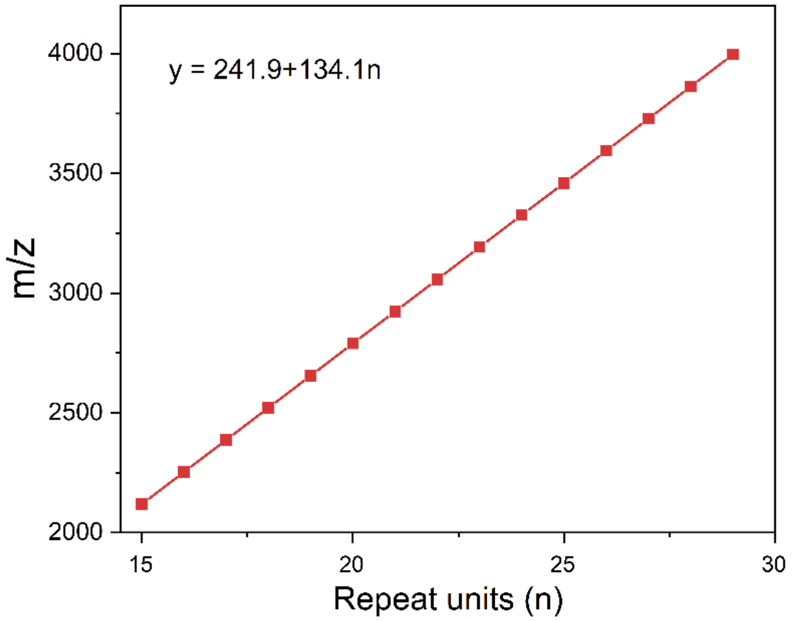

Supplementary Fig. 16. Plot of mass-to-charge ratio (*m/z*) vs number of monomer repeat units from the results of MALDI-TOF analysis of a poly(*p*-MOS) using **2b** as CTA.

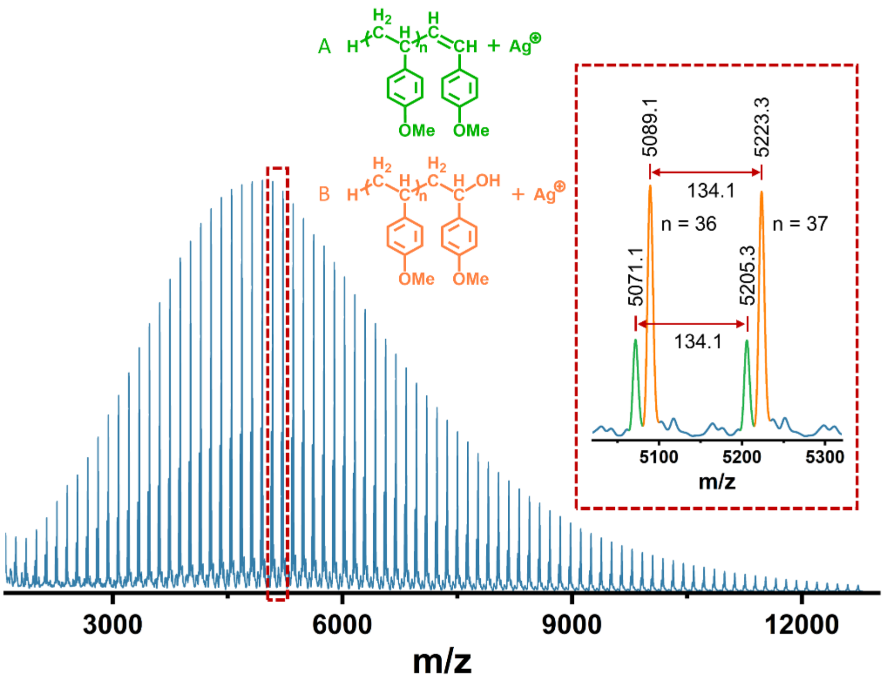

Supplementary Fig. 17. MALDI-TOF spectra of a poly(*p*-MOS) using **2a** as CTA and structural assignment of the polymer, including the most of the chain-end groups were hydrolyzed.

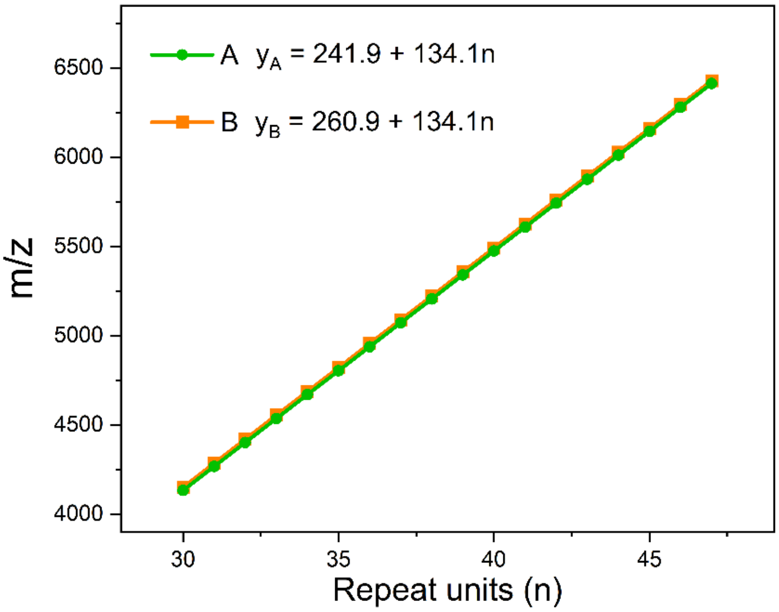

Supplementary Fig. 18. Plot of mass-to-charge ratio (*m/z*) vs. number of monomer repeat units from the results of MALDI-TOF analysis of a poly(*p*-MOS) using **2a** as CTA.

Procedure for Light On-Off Experiments of Photo-controlled Living Cationic Polymerization of 4-Methoxystyrene

In an argon-filled glovebox, 4-Methoxystyrene (0.67 mL, 5.0 mmol, 50.0 equiv.), 0.2 mL of a stock solution of phosphate CTA (**2**) in DCM (0.5 mM, 0.1 mmol, 1.0 equiv.), 0.2 mL of a stock solution of OPC **1** in DCM (0.02 mM, 0.004 mmol, 0.04 equiv.), and 0.4 mL mixed solvent CH_2Cl_2/Et_2O (98/2 vol%) were charged in an oven-dried one-dram vial with a stir bar. A septum cover was then applied to the vial, which was then put outside the glove box in front of a Green LEDs spot lamp (5 W, λ_{max} = 532 nm, 30 mW/cm^2). Aliquot was taken after 5 min for ^{1}H NMR analysis, and then stirred in the dark for the same periods before another aliquot was taken out for ^{1}H NMR and GPC analysis. The same procedures were repeated once more. After that, exposed to light again for 10 min and then stirred in the dark for the same time periods. Aliquots were taken after each period and subjected to ^{1}H NMR and GPC analysis. After that, the reaction was irradiated again for another 20 min. The ^{1}H NMR analysis shown the total monomer conversion was over 60% (Figure 5a) and GPC in different monomer conversion was shown in Figure S15.

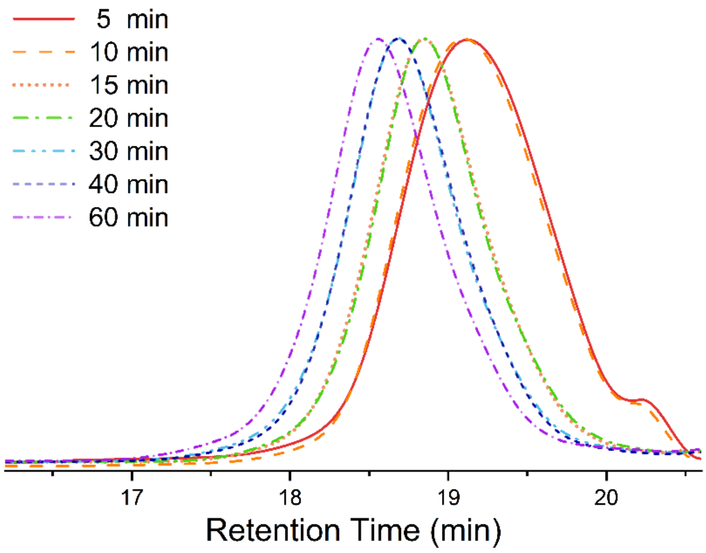

Supplementary Fig. 19. GPC trace of poly(*p*-MOS) obtained in switch on-off measurement.

Prolong on-off experiments: In an argon-filled glovebox, 4-Methoxystyrene (0.67 mL, 5.0 mmol, 50.0 equiv.), 0.2 mL of a stock solution of phosphate CTA (**2**) in DCM (0.5 mM, 0.1 mmol, 1.0 equiv.), 0.2 mL of a stock solution of OPC **1** in DCM (0.02 mM, 0.004 mmol, 0.04 equiv.), and 0.4 mL mixed solvent CH_2Cl_2/Et_2O (98/2 vol%) were charged in an oven-dried one-dram vial with a stir bar. A septum cover was then applied to the vial, which was then put outside the glove box in front of a Green LEDs spot lamp (5 W, λ_{max} = 532 nm, 30 mW/cm^2). Aliquots were taken for ^{1}H NMR analysis at 15 min, 30 min, and the monomer conversion was determined to be 56 %. Then, the reaction was stirred in the dark and aliquots were taken for ^{1}H NMR analysis at 1110 min and 2190 min, which indicated almost no monomer conversion (65%) in this long dark period. Finally, the reaction mixture was exposed to light again, and aliquots were taken at each 15 min interval the final monomer conversion was over 90% (Figure 5b). A prolonged dark period on-off experiment (24 h) was also measured and shown in Figure S16.

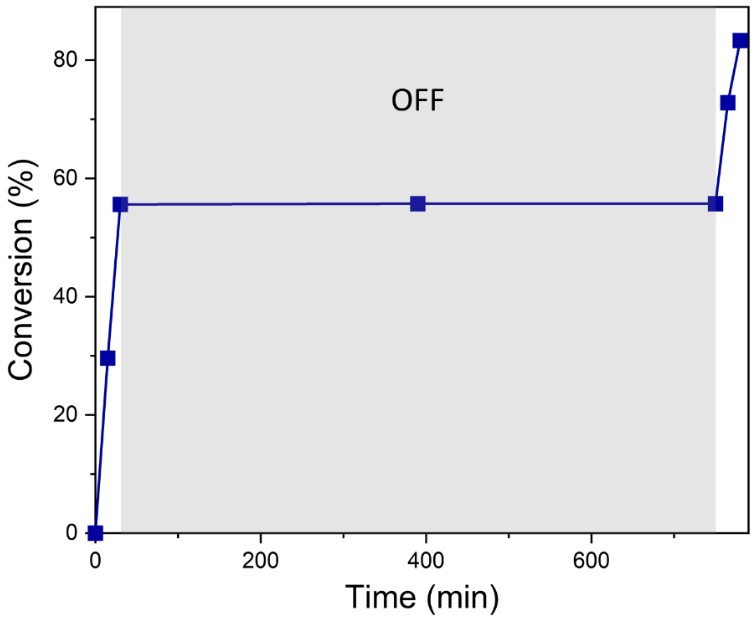

Supplementary Fig. 20. Conversion vs time development of on/off experiment with 24 h prolong off-period using OPC **1** as the photocatalyst and **2a** as the CTA.

methoxyphenyl)methylium tetrafluoroborate as photocatalyst; M_n = 8.1 kg/mol, $Đ$ = 1.31.

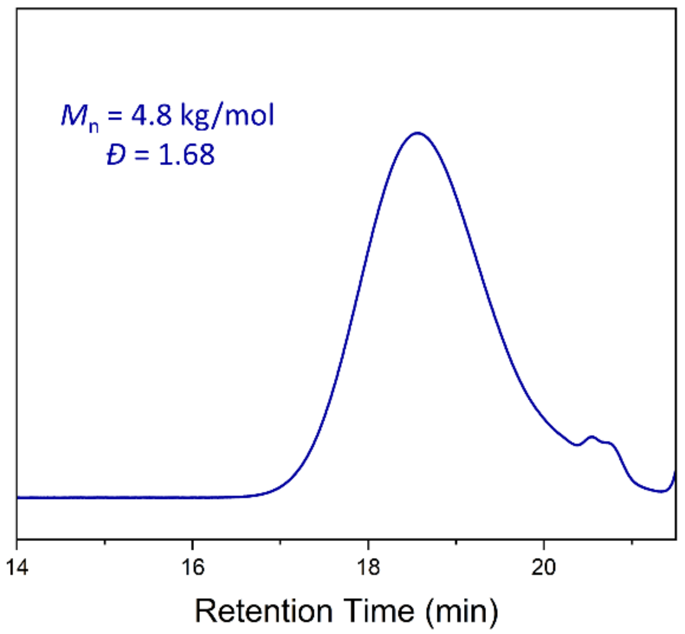

Supplementary Fig. 21. GPC trace of poly(*p*-MOS) using triphenylmethylium tetrafluoroborate as photocatalyst; M_n = 4.8 kg/mol, $Đ$ = 1.68.

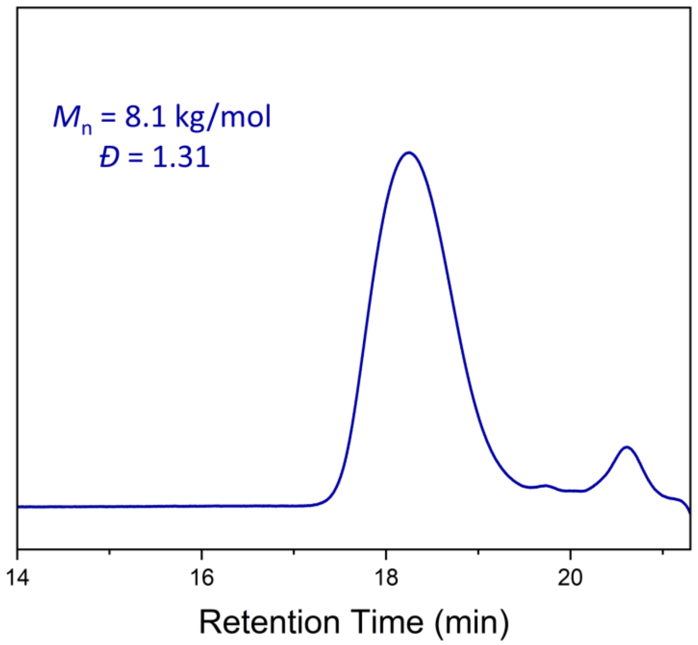

Supplementary Fig. 22. GPC trace of poly(*p*-MOS) using tris(*p*-methoxyphenyl)methylium tetrafluoroborate as photocatalyst; M_n = 8.1 kg/mol, $Đ$ = 1.31.

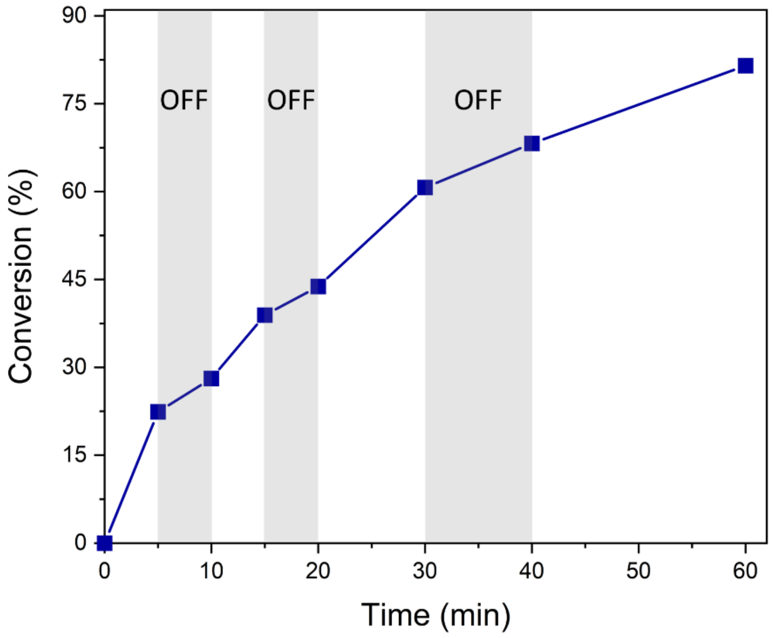

Supplementary Fig. 23. Monomer conversion of *p*-MOS by using tris(*p*-methoxyphenyl)methylium tetrafluoroborate and **2a** with short dark intervals.

Procedure for Cyclic Voltammetry (CV).

All electrochemical measurements were performed on a CHI 760E electrochemistry workstation in a three-electrode system with platinum sheet, calomel electrode (Hg/Hg_2Cl_2), a glassy carbon (0.22 cm^2) as counter electrode, reference electrode, and working electrode, respectively. Using acetonitrile containing 0.10 M tetra-n-butylammonium hexafluorophosphate (TBAH) as a supporting electrolyte, electrodes were immersed in glass flasks under argon at 298 K. The scan rate was set at 50 $mV\cdot s^{-1}$. Oxidation potentials for **2a**, **2b**, *p*-MOS, and **2b** + *p*-MOS were determined at the onset of the oxidation wave, which were conducted for the following mixtures:

1). **2a** (1×10^{-3} M) and TBAH (0.1 M) in MeCN;

2). **2b** (1×10^{-3} M) and TBAH (0.1 M) in MeCN;

3). *p*-MOS (5×10^{-2} M) and TBAH (0.1 M) in MeCN;

4). **2b** (1×10^{-3} M), *p*-MOS (5×10^{-2} M) and TBAH (0.1 M) in MeCN.

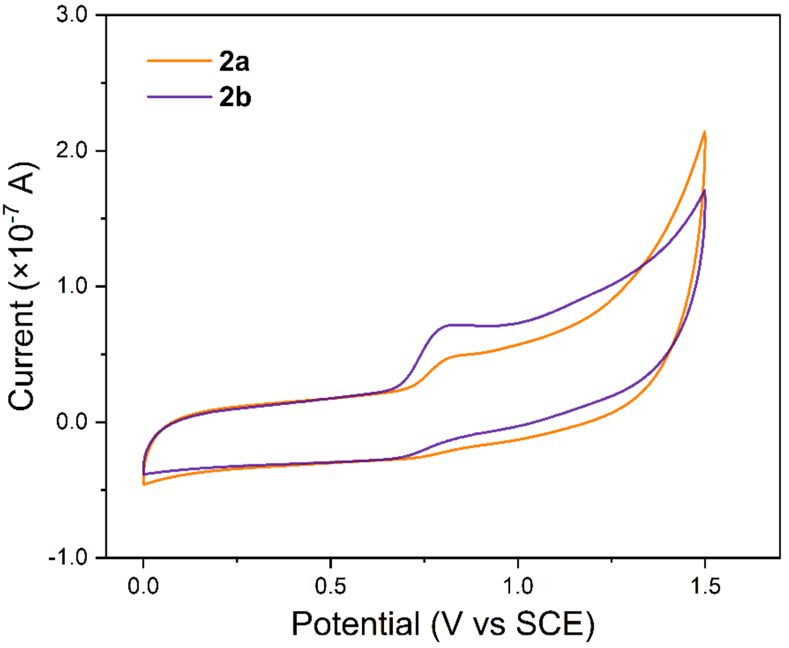

Supplementary Fig. 24. Cyclic voltammogram of **2a** (orange) and **2b** (purple).

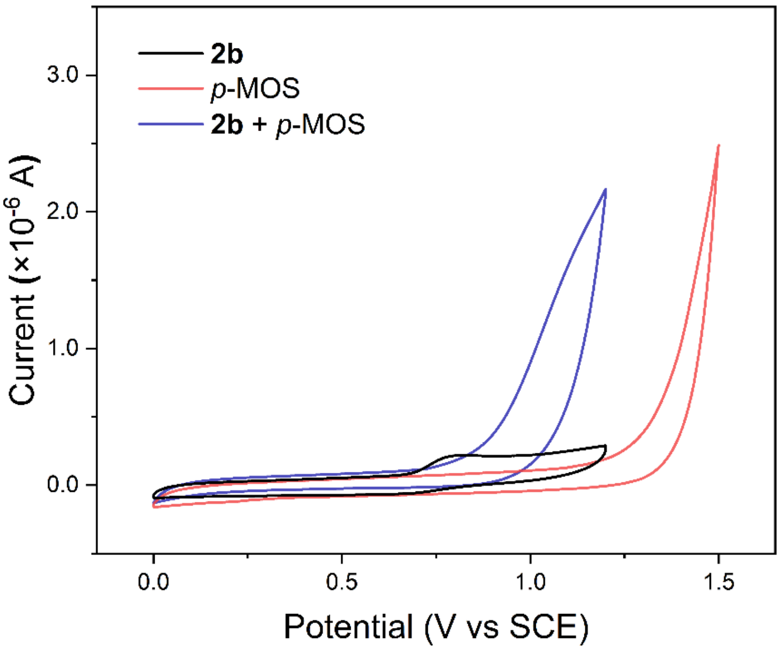

Supplementary Fig. 25. Cyclic voltammogram of **2b** (black), *p*-MOS (red), and **2b** + *p*-MOS (blue).

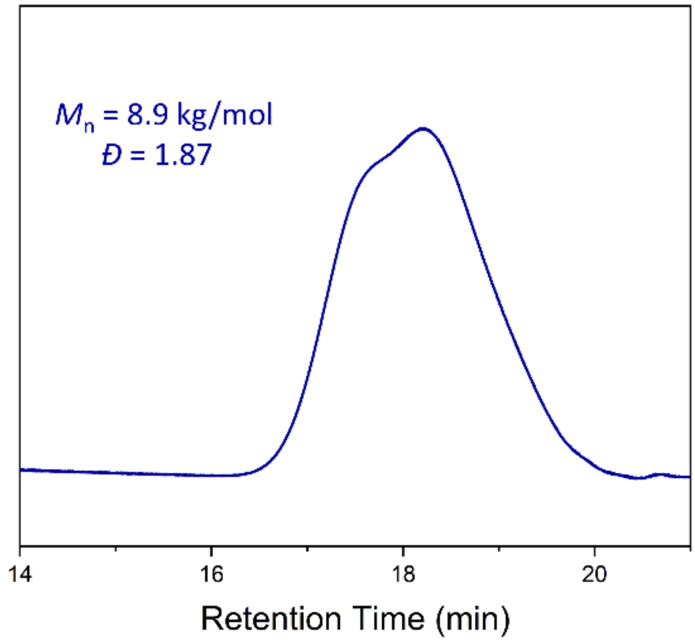

Supplementary Fig. 26. GPC trace of poly(*p*-MOS) prepared by electro-polymerization; M_n = 8.9 kg/mol, *Đ* = 1.87.

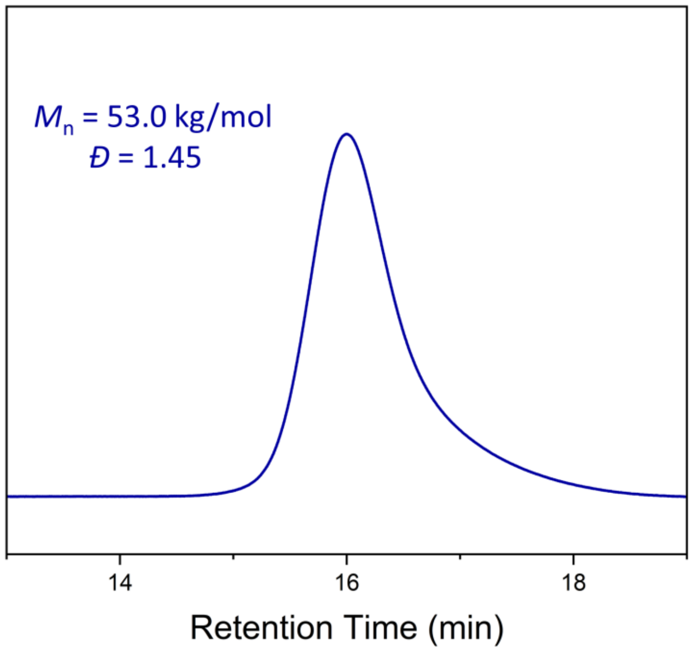

Supplementary Fig. 27. GPC trace of poly(*p*-MOS) at the $[monomer]_0/[CTA]_0$ ratio of 500 :1; M_n = 53.0 kg/mol, *Đ* = 1.45

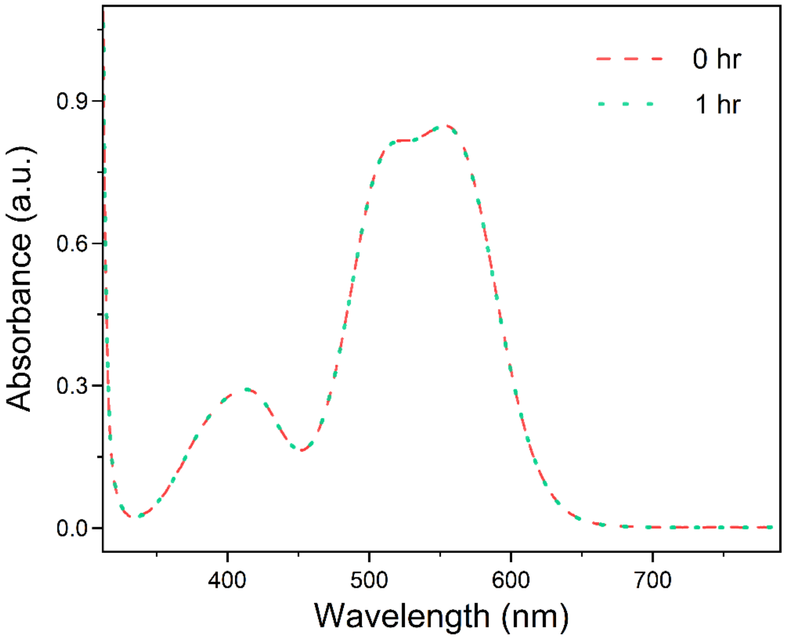

Supplementary Fig. 28. UV/vis absorption spectrum of the polymerization mixture before and after photo-polymerization for 1 h.

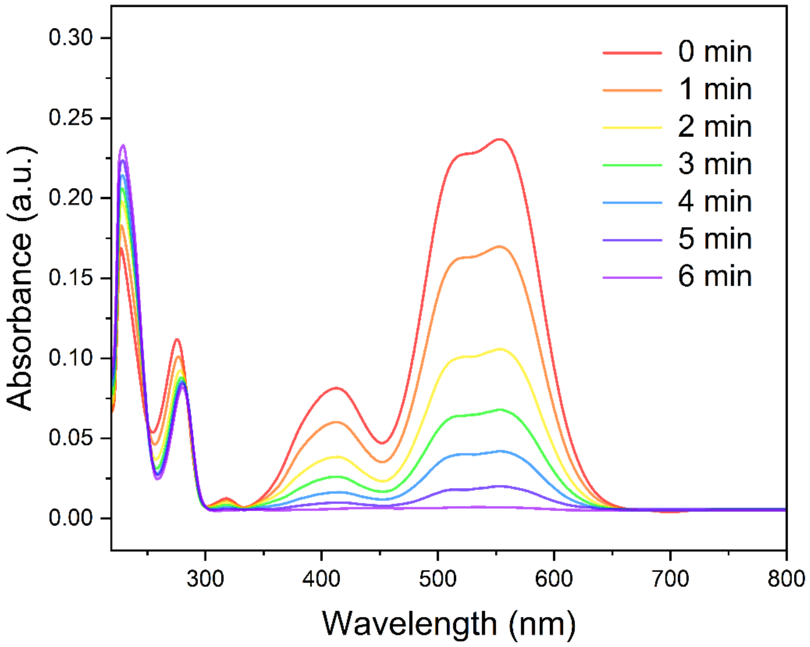

Supplementary Fig. 29. UV/vis absorption spectrum of the 2 mL DCM solution of OPC **1** (5×10^{-6} M) after addition of 5 μL sodium hydroxide (0.01 M) solution in methanol.

Theoretical Calculation

Geometry optimization and total energy calculation of all these structures were performed with the Gaussian09 A01[1] at B3LYP/6-31G* without any symmetry restriction.[2-4] In the meanwhile, the PCM solvation model was used to deal with the solvation effect. In the PCM method, the solvent was treated as an infinite continuum dielectric and the solvent response to the presence of the electric field generated by the solute charge distribution (assumed to be contained in a volume of known form and dimension, the molecular cavity) was represented in terms of an apparent charge spread on the cavity surface. Here, all the calculations were carried out in dichloromethane phase unless otherwise specified. After the geometry optimization was performed, analytical vibration frequencies were calculated at the same level to determine the nature of the located stationary point. The visualization of molecular orbitals is achieved through Multiwfn[5] and vmd1.9.3[6].

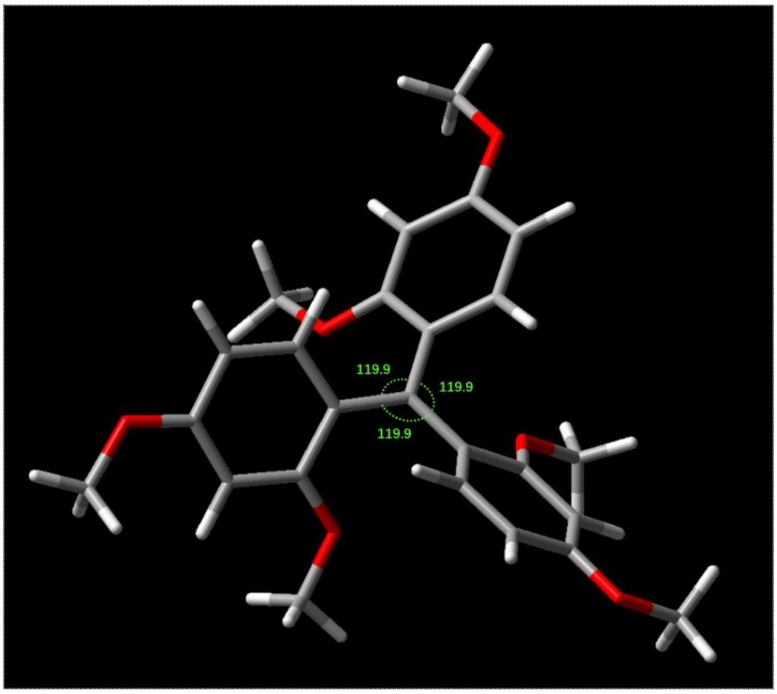

Supplementary Fig. 30. Optimized chemical conformation of OPC **1**, which indicates the approximate planer of three bonds link to central carbon atom.

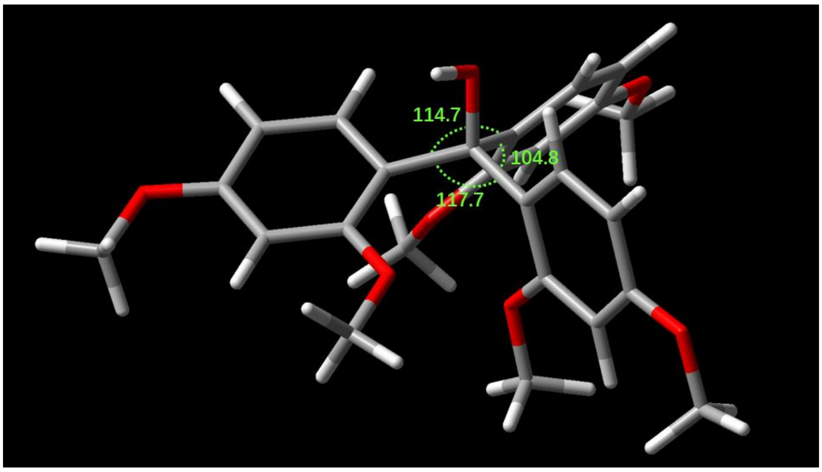

Supplementary Fig. 31. Optimized chemical conformation of **1-OH**, which indicates the approximate tetrahedrold of three bonds link to central carbon atom.

3. Supplementary Table 1

Supplementary Table 1. Comparison of the redox potentials of triarylmethyl cations

Triarylmethyl Cations	Counterion	$E_{PC+*/PC}$[a] (V vs SCE)	$E_{PC+/PC}$ (V vs SCE)
Ph_3C^+	BF_4^-	+2.68	0.38
$(p\text{-}MeOC_6H_4)Ph_2C^+$	BF_4^-	+2.46	0.18
$(p\text{-}MeOC_6H_4)_2PhC^+$	BF_4^-	+2.33	0.05
$(p\text{-}MeOC_6H_4)_3C^+$	BF_4^-	+2.15	-0.21
OPC **1**	BF_4^-	+1.55	-0.36

[a]Oxidation potentials of the excited triarylmethyl cations calculatcd according to Eq. 1.

4. Supplementary References

(1) Gaussian 09, revision D. 01, Frisch, M. J.; Trucks, G. W.; Schlegel, H. B.; Scuseria, G. E.; Robb, M. A.; Cheeseman, J. R.;Scalmani, G.; Barone, V.; Mennucci, B.; Petersson, G. A.; Nakatsuji, H.; Caricato, M.; Li, X.; Hratchian, H. P.; Izmaylov, A. F.; Bloino, J.; Zheng, G.; Sonnenberg, J. L.; Hada, M.; Ehara, M.; Toyota, K.; Fukuda, R.; Hasegawa, J.; Ishida, M.; Nakajima, T.; Honda, Y.; Kitao, O.; Nakai, H.; Vreven, T.; Montgomery, J.; Peralta, J. A. J. E.; Ogliaro, F.; Bearpark, M.; Heyd, J. J.; Brothers, E.; Kudin, K. N.; Staroverov, V. N.; Kobayashi, R.; Normand, J.; Raghavachari, K.; Rendell, A.; Burant, J. C.; Iyengar, S. S.; Tomasi, J.; Cossi, M.; Rega, N.; Millam, N. J.; Klene, M.; Knox, J. E.; Cross, J. B.; Bakken, V.; Adamo, C.; Jaramillo, J.; Gomperts, R.; Stratmann, R. E.; Yazyev, O.; Austin, A. J.; Cammi, R.; Pomelli, C.; Ochterski, J. W.; Martin, R. L.; Morokuma, K.; Zakrzewski, V. G.; Voth, G. A.; Salvador, P.; Dannenberg, J. J.; Dapprich, S.; Daniels, A. D.; Farkas, O.; Foresman, J. B.; Ortiz, J. V.; Cioslowski, J.; Fox, D. J. Gaussian, Inc. Wallingford CT, **2013**.

(2) Vosko, S. H.; Wilk, L.; Nusair, M. Accurate spin-dependent electron liquid correlation energies for local spin density calculations: a critical analysis. *Can. J. Phys*. **1980**, *58*, 1200.

(3) Becke, A. D. Density-Functional Thermochemistry. V. Systematic optimization of exchange-correlation functionals. *J. Chem. Phys*. **1997**, *107,* 8554.

(4) Kohn, W.; Becke, A.D.; Parr, R.G. Density functional theory of electronic structure. *J. Phys. Chem.* **1996**, *100*, 12974.

(5) Lu, T.; Chen, F. W. Multiwfn: A multifunctional wavefunction analyzer. *J. Comput. Chem*. **2012**, *33*, 580.

(6) Humphrey, W.; Dalke, A.; Schulten, K. VMD–visual molecular dynamics. *J. Molec. Graphics* **1996**, *14*, 33.
